# Supplementary material for: Modeling protein–small molecule conformational ensembles with PLACER
Source: Proc Natl Acad Sci U S A. 2025 Nov 4;122(45):e2427161122. doi: 10.1073/pnas.2427161122 (PMC12625923; doi:10.1073/pnas.2427161122)
Supplement: Supplementary file 1 [file pnas.2427161122.sapp.pdf]

Supporting Information

Modeling protein-small molecule conformational ensembles with PLACER

Ivan Anishchenko[1,2†], Yakov Kipnis[1,2,4†], Indrek Kalvet[1,2,4†], Guangfeng Zhou[1,2], Rohith Krishna[1,2], Samuel J. Pellock[1,2], Anna Lauko[1,2,3], Gyu Rie Lee[1,2,4], Linna An[1,2], Justas Dauparas[1,2], Frank DiMaio[1,2], David Baker[1,2,4*]

†These authors contributed equally
*To whom correspondence should be addressed: dabaker@uw.edu
1. Department of Biochemistry, University of Washington, Seattle, WA 98105, USA
2. Institute for Protein Design, University of Washington, Seattle, WA 98105, USA
3. Graduate Program in Biological Physics, Structure and Design, University of Washington, Seattle, WA 98105, USA
4. Howard Hughes Medical Institute, University of Washington, Seattle, WA 98105, USA

1. PLACER model training and architecture 2
   1.1. Training on the PDB 2
   1.2. Training on the CSD 3
   1.3. Network input features 3
   1.4. PLACER architecture 5
   1.5. Losses 6
   1.6. List of algorithms 8
   1.7. Analysis of potential data leakage for training on the CSD 12
2. Astex non-native benchmark 15
3. PoseBusters benchmark 18
4. Computational analysis of RA95 series of designed and evolved retroaldolases 22
5. Computational de novo design of retroaldolases in NTF2 fold 23
   5.1. Identification of focus theozyme configuration 25
   5.2. Application of PLACER for design selection 26
   5.3. IVTT based retroaldolase activity testing assay 27
   5.4. Characterization of individual variants 27
   5.5. In depth PLACER analysis of the experimentally tested designs 28
6. References 30

1. PLACER model training and architecture

1.1. Training on the PDB

Training and validation sets were compiled from structures deposited to the Protein Data Bank (1) before January 12, 2023. We selected X-ray and EM structures with resolution 2.5Å and better and filtered out entries having ≥10% of unresolved atoms, as well as entries with ≥20 non-standard residues across all polypeptide chains in the asymmetric unit. These filters reduced the size of the set by 33% from 200,713 to 135,172. We additionally removed entries which share similarity to the test set (we used Astex Non-Native Set (2) as our primary small molecule docking benchmark set) either on the protein or the small molecule levels. A PDB structure was discarded if any of its protein chains had ≥30% sequence identity to any of the protein chains in the test set. Structures containing small molecules with ≥80% Tanimoto similarity to the small molecules in the test set were also excluded. We used Open Babel (3) to first compute the small molecule FP4 fingerprints and then to calculate the Tanimoto coefficients. The remaining 119,916 PDB structures were randomly split into training (112,828) and validation (7,090) sets such that none of the protein chains in the two sets share >30% sequence identity. For every structure in the training and validation sets, we counted the number of entries within the set which share ≥30% sequence identity on the protein chain level. The inverse of these counts were then used during training and validation to sample the structures from the respective set.

During training and validation, we do not split the PDB entries into individual chains or molecules but operate on the whole asymmetric units. We directly parse PDBx/mmCIF files keeping all molecular content; only water molecules are excluded. The Chemical Component Dictionary (4) is used to construct chemical graphs for individual residues and small molecules observed in a PDB entry; nodes in a chemical graph represent individual atoms (only non-hydrogen atoms are considered) and edges represent chemical bonds between atoms. The residue-level graphs are joined together into a single chemical graph for the whole PDB entry. Edges are added to connect subgraphs representing individual residues within a polymer chain (proteins, DNA, RNA), as well as are edges parsed from the `struct_conn` data records of the PDBx/mmCIF file. Atomic coordinates are parsed from the PDBx/mmCIF file and are saved on a corresponding node of the chemical graph.

PDB structures were cropped to at most 600 heavy atoms to fit the GPU memory. First, we split all atoms in the input structure into four classes (protein, nucleic acid, small molecule, metal), then randomly choose one of these classes with ratios 1:1:5:1, and finally sample a random heavy atom from the selected class to be the center of the cropped region. The crop is defined as the collection of residues and small molecules in the PDB structure which are closest in space to the residue or the small molecule the crop center belongs to. Atom coordinates in the crop undergo corruption. First, we collect all atoms in the 8-hop neighborhood of the crop center in the chemical graph and add them to the corruption set. Backbone atoms in polypeptide (N,CA,C), polyribo- and polydeoxyribonucleotide chains (O3', C3', C4', C5', O5', P) which are not part of the corruption set are kept fixed with only a minor Gaussian noise (σ = 0.1Å) added to the atom coordinates to make the network more robust to small displacements in atomic coordinates of the backbone atoms(5). All the remaining non-backbone atoms are included in the corruption set. The chemical graph corresponding to the crop is then split into connected components. If a connected component has fixed backbone atoms, then the corrupted atoms in that component are initialized with the coordinates of the closest backbone atom in the graph (based on the shortest path in the graph). Otherwise, a random atom

is picked from the component, Gaussian noise with σ = 1.5Å is added to its coordinates, and all other atoms reachable from the selected atom are collapsed to it. Finally, Gaussian noise with σ = 1.5Å is applied to all atoms in the corruption set.

1.2. Training on the CSD

The PDB-derived datasets were complemented by the crystal structures of small molecules deposited to the Cambridge Structural Database (CSD v5.43; November 2021)(6). We used structures with R-factor 7.5% and better and no disorder and excluded polymeric and organo-metallic entries; the number of heavy atoms per small molecule was limited to the 3..80 range. Only molecules which could be successfully parsed by Open Babel were retained. CSD structures with multiple molecules in the asymmetric unit were split into individual molecules, followed by merging of identical molecules with the same canonical SMILES string into one entry for training and validation. Upon merging, we kept 3D coordinates of all the instances to account for alternative conformations during training. Training and validation splits were done using 75% Tanimoto similarity cutoff, yielding 226,684 and 7,116 examples, respectively. During training, molecules were sampled with frequencies inversely proportional to the number of similar molecules in the set at Tanimoto similarity 75%.

1.3. Network input features

Training examples from both the PDB and the CSD were featurized in the same way. The information about atom and bond types which were used as inputs to PLACER is summarized in Table S1. In addition to atom single (`f1d`) and pair (`f2d`) features, PLACER also utilizes information about the local topology of the input molecules which is represented by bonding, bond length, chirality and planarity terms (for atoms which are immediately connected in the chemical graph, Table S1, entries 18-21).

Table S1. Description of PLACER input features.

Entry	Input name (Shape)	Description
1	`xyz` (N_{atoms}, 3)	initial atomic coordinates
2	`group` (N_{atoms}, 7)	element's group in the periodic table, 1-hot encoded
3	`period` (N_{atoms}, 18)	element's period in the periodic table, 1-hot encoded
4	`is_lanthanide` (N_{atoms}, 1)	a boolean flag indicating whether the element is a lanthanide
5	`is_actinide` (N_{atoms}, 1)	a boolean flag indicating whether the element is an actinide
6	`lanthanide_group` (N_{atoms}, 15)	element's group within lanthanides, 1-hot encoded
7	`actinide_group` (N_{atoms}, 15)	element's group within actinides, 1-hot encoded

8	**charge**<br>(N_{atoms}, 7)	total charge of the atom, 1-hot encoded from set {-3,-2,-1,0,1,2,3}
9	**nhyd**<br>(N_{atoms}, 4)	number of hydrogens connected to the atom, 1-hot encoded
10	**hyb**<br>(N_{atoms}, 6)	hybridization state of the atom, 1-hot encoded from set {*sp*, sp^2, sp^3, square planar, trigonal bipyramidal, octahedral}
11	**is_corrupted**<br>(N_{atoms}, 1)	a boolean flag indicating whether the atom belongs to the corruption set
12	**f1d**<br>(N_{atoms}, 75)	concatenation of **group**, **period**, **is_lanthanide**, **is_actinide**, **lanthanide_group**, **actinide_group**, **charge**, **nhyd**, **hyb**, **is_corrupted**
13	**is_aromatic**<br>(N_{atoms}, N_{atoms}, 2)	a boolean flag indicating whether the bond is aromatic
14	**is_in_ring**<br>(N_{atoms}, N_{atoms}, 2)	a boolean flag indicating whether the bond is in a ring
15	**bond_order**<br>(N_{atoms}, N_{atoms}, 4)	bond order, 1-hot encoded from set {single, double, triple, other}
16	**bond_separation**<br>(N_{atoms}, N_{atoms}, 7)	an integer counting the number of covalent bonds between any two atoms in the chemical graph, 1-hot encoded. If number of bonds between atoms >7, then separation=0.
17	**f2d**<br>(N_{atoms}, N_{atoms}, 15)	concatenation of **is_aromatic**, **is_in_ring**, **bond_order**, **bond_separation**
18	S_{bonds}<br>(N_{bonds}, 2)	pairs of atoms connected by a chemical bond
19	**bond_len**<br>(N_{bonds})	equilibrium bond lengths for bonds in S_{bonds} based on the covalent radii and bond order (from Open Babel)
20	$S_{chirals}$<br>($N_{chirals}$, 4)	(o,i,j,k) tuples where o is the index of the central pyramidal or tetrahedral sp^3 hybridized atom and its neighboring atoms i,j,k ordered clockwise
21	$S_{planars}$<br>($N_{planars}$, 4)	(o,i,j,k) tuples for planar geometries around an sp^2 hybridized atom o and its neighboring atoms i,j,k

1.4. PLACER architecture

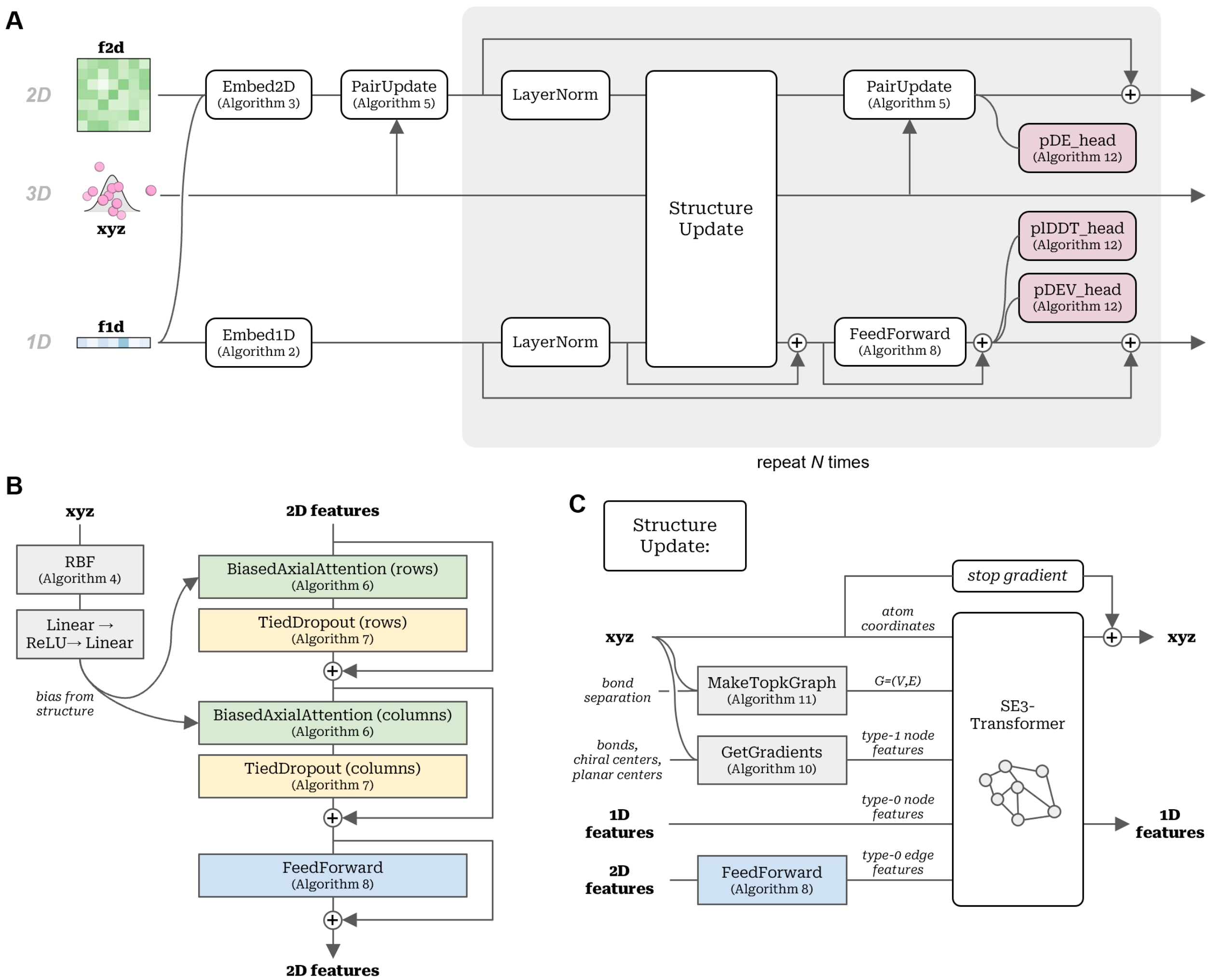

Figure S1. **PLACER architecture**. **A)** The three-track network schematics (Algorithm 1). **B)** PairUpdate module (Algorithm 5). **C)** Structure update module.

1.5. Losses

We use a combination of structure losses and confidence prediction losses to train PLACER:

$$L = w_{aaFAPE}L_{aaFAPE} + w_{aaFAPE}^{interaction}L_{aaFAPE}^{interaction} + w_{aaFAPE}^{small\ molecule}L_{aaFAPE}^{small\ molecule} + w_{bond}L_{bond} + w_{angle}L_{angle} + w_{chiral}L_{chiral} + w_{planar}L_{planar} + w_{plDDT}L_{plDDT} + w_{pDE}L_{pDE} + w_{pRMSD}L_{pRMSD} \quad (1)$$

Table S2. Loss weights used during PLACER training.

training stage	w_{aaFAPE}	$w_{aaFAPE}^{interaction}$	$w_{aaFAPE}^{small\ molecule}$	w_{bond}	w_{angle}	w_{chiral}	w_{planar}	w_{plDDT}	w_{pDE}	w_{pRMSD}
1	1.0	0.0	1.0	0.0	0.0	0.0	0.0	1.0	1.0	1.0
2	1.0	1.0	1.0	1.0	1.0	1.0	1.0	1.0	1.0	1.0

All-atom FAPE loss L_{aaFAPE} is a modification of the original FAPE loss introduced in Ref. (7) allowing to operate on molecules of any type and to treat the entire molecule flexibly (no need to define rigid groups, like backbone N-Ca-C in the original FAPE). First, we enumerate all triples i, j, k of atoms which are covalently bonded (there are covalent bonds between atoms i, j and j, k). Spatial coordinates $\vec{x}_i$,$\vec{x}_j$,$\vec{x}_k$ of these three atoms in the predicted structure are used to construct a local reference frame according to Eqs. 2:

$$\begin{aligned} \vec{u} &= \vec{x}_j - \vec{x}_i \\ \vec{v} &= \vec{x}_k - \vec{x}_j \\ \vec{e}_1 &= \frac{\vec{u}\times\vec{v}}{|\vec{u}\times\vec{v}|},\ \vec{e}_3 = \frac{\vec{u}-\vec{v}}{|\vec{u}-\vec{v}|},\ \vec{e}_2 = \vec{e}_3 \times \vec{e}_1 \\ R_{(ijk)}^{x} &= [\vec{x}_i, \vec{x}_j, \vec{x}_k] \end{aligned} \quad (2)$$

Similarly, a frame $R_{(ijk)}^{y}$ is constructed from the atom coordinates in the reference experimental structure. The two frames $R_{(ijk)}^{x}$, $R_{(ijk)}^{y}$ and the two sets of atom coordinates – one in the model structure $\{\vec{x}\}$ and one in the reference $\{\vec{y}\}$ – are then used to calculate deviations in atom positions once the frames are aligned:

$d_{(ijk),l} = \left|R_{(ijk)}^{x}(\vec{x}_l - \vec{x}_j) - R_{(ijk)}^{y}(\vec{y}_l - \vec{y}_j)\right|$ – deviation in positions for atom l

$$L_{aaFAPE} = \frac{1}{N_{atoms}N_{frames}} \sum_{(ijk)\in S_{frames}} \sum_{l=1}^{N_{atoms}} d_{(ijk),l}$$

To put more weight during training on the small molecule, and the interaction between the small molecule and the rest of the structure, we also used two slightly modified versions of all-atom FAPE with ${L_{aaFAPE}}^{p,sm}$ measuring deviations of small molecule atoms in the protein frames and ${L_{aaFAPE}}^{sm,sm}$ emphasizing the internal structure of the small molecule.

$${L_{aaFAPE}}^{interaction} = L_{aaFAPE}(S_{frames} \in protein, l \in small\ molecule)$$
$${L_{aaFAPE}}^{small\ molecule} = (S_{frames} \in small\ molecule, l \in small\ molecule)$$

Bonded losses. Bond loss term L_{bond} measures the distance deviation for covalently bonded atoms between the model and the reference structures:

$$L_{bond} = MSE_{(ij)\in S_{bonds}}\left(\left|\vec{x_i} - \vec{x_j}\right|, \left|\vec{y_i} - \vec{y_j}\right|\right)$$

where MSE is a mean squared error loss. MSE loss is also used to measure deviations in bonded angles:

$$L_{angle} = MSE_{(ijk)\in S_{angles}}\left(\alpha^{x}_{(ijk)}, \alpha^{y}_{(ijk)}\right)$$

where $\alpha^{x}_{(ijk)}$ and $\alpha^{y}_{(ijk)}$ are planar angles (in radians) between atoms i, j, k calculated from the model and the reference structures respectively.

Additional loss term L_{chiral} is imposed on all quadruples of atoms $o, i, j, k \in S_{chirals}$ where the three atoms i, j, k are covalently bonded to an sp^3-hybridized central atom o to force the off-plane geometry. This is achieved by first computing the angle $\theta_{(oijk)}$ between the normal to the o, i, j plane $\vec{n}_{(oij)} = (\vec{x_i} - \vec{x_o}) \times (\vec{x_j} - \vec{x_o})$ and the off-plane vector $\vec{v}_{(ok)} = \vec{x_k} - \vec{x_o}$ and then applying the MSE loss:

$$L_{chiral} = MSE_{(oijk)\in S_{chirals}}\left(\theta^{x}_{(oijk)}, \theta^{y}_{(oijk)}\right)$$

A similar loss term L_{planar} is used to enforce planar geometry around the sp^2-hybridized atom o and its neighboring atoms i, j, k:

$$L_{planar} = MSE_{(oijk)\in S_{planars}}\left(\theta^{x}_{(oijk)}, \theta^{y}_{(oijk)}\right)$$

Confidence losses. We model per-atom confidences in two different ways. One is through predicted lDDT (plDDT) score which has previously been introduced in the context of the AF2 network(7); for PLACER we extended it to the all atom representation. Additionally, we predict uncertainties in atomic positions through estimating the expected standard deviations between atom x, y, z coordinates in the model and in the reference structures. These standard deviations, if combined over the entire structure or a region of interest, give rise to the predicted RMSD (pRMSD) score (see Main text for details). Lastly, to estimate atom-atom pair accuracies, we predicted errors in distances between every two atoms. All-by-all distance matrix of the model was subtracted from the one of the reference to get the signed distance error matrix (referred to as "estograms").(8) This matrix was then binned into 102 bins with 100 equal bins covering the [-5;5] interval plus the two bins corresponding to the outside regions (-∞;-5) and (5;∞)). The network was trained to make categorical predictions over these bins for which the categorical cross-entropy loss was used during training.

1.6. List of algorithms

Algorithm 1. PLACER network

def PLACER($\mathbf{X}_i$, $\mathbf{f1d}_i$, $\mathbf{f2d}_{i,j}$, separation, bonds, bondlen, chirals, planars, N_{iter}):
# embed inputs
  $\mathbf{single}_i^{prev}$ = Embed1D($\mathbf{f1d}_i$, c = 64) *# Algorithm 2,* $\mathbf{single}_i \in \mathbb{R}^{64}$
  $\mathbf{pair}_{i,j}^{prev}$ = Embed2D($\mathbf{f2d}_{i,j}$, c = 128) *# Algorithm 3,* $\mathbf{pair}_{i,j} \in \mathbb{R}^{128}$
  $\mathbf{pair}_{i,j}^{prev}$ = PairUpdate($\mathbf{pair}_{i,j}^{prev}$, $\mathbf{X}_i$, c = 32, N_{head} = 4, p_{drop} = 0.15) *# Algorithm 5*
# iteratively update coordinates and 1D and 2D embeddings
  Xs, plDDTs, pDEVs, pDEs = [$\mathbf{X}_i$], [ ], [ ], [ ] *# lists for saving results*
  scale_factor = 100.0
  for iter = 1 ... N_{iter}: *# network weights are shared within this block*
    $\mathbf{single}_i$ = LayerNorm($\mathbf{single}_i^{prev}$) **if** iter > 1 **else** $\mathbf{single}_i^{prev}$
    $\mathbf{pair}_{i,j}$ = LayerNorm($\mathbf{pair}_{i,j}^{prev}$) **if** iter > 1 **else** $\mathbf{pair}_{i,j}^{prev}$
    $\mathbf{g}_i$ = GetGradients(Xs[iter], bonds, bondlen, chirals, planars) *# Algorithm 10,* $\mathbf{g}_i \in \mathbb{R}^{3,3}$
    $\boldsymbol{G}$ = MakeTopkGraph(Xs[iter], separation, topk = 32) *# Algorithm 11*
    $\mathbf{edge\_features}_k$ = FeedForward($\mathbf{pair}_{i,j \in \boldsymbol{G}.\mathbf{edges}}$, normalize=False) *# Algorithm 8*
    $\mathbf{state}_i$, $\mathbf{dx}_i$ = SE3_Transformer($\boldsymbol{G}$, $\mathbf{single}_i$, $\mathbf{l1}_i$, $\mathbf{edges}_k$)
# update coords and embeddings
    $\mathbf{X}_i$ = stopgrad($\mathbf{X}_i$) + $\mathbf{dx}_i$ / *scale_factor*
    $\mathbf{single}_i$ = UpdateSingle($\mathbf{single}_i$ + $\mathbf{state}_i$) *# FeedForward(x,c=128,n=2,pdrop=0.15,normalize=True)*
    $\mathbf{pair}_{i,j}$ = PairUpdate($\mathbf{pair}_{i,j}$, $\mathbf{X}_i$, c = 32, N_{head} = 4, p_{drop} = 0.15)
# auxiliary heads
    $\mathbf{plddt}_i$ = PredictError($\mathbf{single}_i$, bins=51) *# Algorithm 12*
    $\mathbf{dev}_i$ = PredictError($\mathbf{single}_i$, bins=1) *# Algorithm 12*
    $\mathbf{pde}_{i,j}$ = PredictError($\mathbf{pair}_{i,j}$+$\mathbf{pair}_{j,i}$, bins=102) *# Algorithm 12*
# residual connections
    $\mathbf{single}_i^{prev}$ += $\mathbf{single}_i$
    $\mathbf{pair}_{i,j}^{prev}$ += $\mathbf{pair}_{i,j}$
# append predictions from the current iteration to the output lists
    Xs += [$\mathbf{X}_i$]
    plDDTs += [$\mathbf{plddt}_i$]
    pDEVs += [$\mathbf{dev}_i$]
    pDEs += [$\mathbf{pde}_{i,j}$]
  return Xs, pDEs, plDDTs, pDEVs

Algorithm 2. Initial embedding of node features

def Embed1D($\mathbf{f1d}_i$, c = 64):
  $\mathbf{s}_i$ = Linear($\mathbf{f1d}_i$) # $\mathbf{s}_i \in \mathbb{R}^c$
  $\mathbf{s}_i$ = LayerNorm(Linear(ReLU($\mathbf{s}_i$)))
  return $\mathbf{s}_i$

Algorithm 3. Initial embedding of pair features

def Embed2D($\mathbf{f1d}_i$, $\mathbf{f2d}_{i,j}$, c = 128):
$\mathbf{s}_i^{left}$, $\mathbf{s}_i^{right}$ = Linear($\mathbf{f1d}_i$) # $\mathbf{s}_i^{left}, \mathbf{s}_i^{right} \in \mathbb{R}^c$
$\mathbf{p}_{i,j}$ = Linear($\mathbf{f2d}_{i,j}$) # $\mathbf{p}_{i,j} \in \mathbb{R}^c$
$\mathbf{s}_{i,j} = \mathbf{s}_i^{left} + \mathbf{s}_j^{right}$ # *outer summation*
$\mathbf{p}_{i,j}$ = concat($\mathbf{p}_{i,j}$, $\mathbf{s}_{i,j}$) # $\mathbf{p}_{i,j} \in \mathbb{R}^{2c}$
$\mathbf{p}_{i,j}$ = ReLU($\mathbf{p}_{i,j}$)
$\mathbf{p}_{i,j}$ = Linear($\mathbf{p}_{i,j}$) # $\mathbf{p}_{i,j} \in \mathbb{R}^c$
$\mathbf{p}_{i,j}$ = LayerNorm($\mathbf{p}_{i,j}$)
return $\mathbf{p}_{i,j}$

Algorithm 4. Project pairwise distances for a set of points onto Gaussian radial basis functions

def RBF($\mathbf{X}_i$, N_{rbf} = 32, D_{max} = 20.0):
$D_{i,j} = \|\mathbf{X}_i - \mathbf{X}_j\|$
$\sigma = D_{max} / N_{rbf}$
$\mathbf{rbf}_{i,j}$ = concat(exp($-((D_{i,j} - k\sigma)/\sigma)^2$) **for** k **in** $1,\ldots,N_{rbf}$) # $\mathbf{rbf}_{i,j} \in \mathbb{R}^{Nrbf}$
return $\mathbf{rbf}_{i,j}$

Algorithm 5. Pair update with bias from structure

def PairUpdate($\mathbf{pair}_{i,j}$, $\mathbf{xyz}_i$, c = 32, N_{head} = 4, p_{drop} = 0.15):
$\mathbf{rbf}_{i,j}$ = RBF($\mathbf{xyz}_i$, $N_{rbf} = c$) # *Algorithm 4*, $\mathbf{rfb}_{i,j} \in \mathbb{R}^c$
$\mathbf{bias}_{i,j}$ = Linear($\mathbf{rbf}_{i,j}$) # $\mathbf{bias}_{i,j} \in \mathbb{R}^c$
$\mathbf{bias}_{i,j}$ = Linear(ReLU($\mathbf{bias}_{i,j}$)) # $\mathbf{bias}_{i,j} \in \mathbb{R}^{cn}$
attention and dropout applied to rows
$\mathbf{pair}_{i,j}^{row}$ = BiasedAxialAttention($\mathbf{pair}_{i,j}$, $\mathbf{bias}_{i,j}$, c, N_{head}, is_row = *True*) # $\mathbf{pair}_{i,j}^{row} \in \mathbb{R}^{cn}$
$\mathbf{pair}_{i,j}^{row}$ = TiedDropout($\mathbf{pair}_{i,j}^{row}$, p_{drop}, is_row = *True*)
$\mathbf{pair}_{i,j}$ += $\mathbf{pair}_{i,j}^{row}$
attention and dropout applied to columns
$\mathbf{pair}_{i,j}^{col}$ = BiasedAxialAttention($\mathbf{pair}_{i,j}$, $\mathbf{bias}_{i,j}$, c, N_{head}, is_row = *False*) # $\mathbf{pair}_{i,j}^{col} \in \mathbb{R}^{cn}$
$\mathbf{pair}_{i,j}^{col}$ = TiedDropout($\mathbf{pair}_{i,j}^{col}$, p_{drop}, is_row = *False*)
$\mathbf{pair}_{i,j}$ += $\mathbf{pair}_{i,j}^{col}$
feed-forward layer
$\mathbf{pair}_{i,j}$ += FeedForward($\mathbf{pair}_{i,j}$, p_{drop}) # $\mathbf{pair}_{i,j} \in \mathbb{R}^{cn}$
return $\mathbf{pair}_{i,j}$

Algorithm 6. Tied axial attention with bias from structure

```
def BiasedAxialAttention(pair_i,j, bias_i,j, c, N_head, is_row = True):
    if is_row is True:                                    # flip rows and columns
        pair_i,j = pair_j,i
        bias_i,j = bias_j,i
    pair_i,j = LayerNorm(pair_i,j)
    bias_i,j^h = Linear(LayerNorm(bias_i,j))              # bias_i,j^h ∈ ℝ, h = 1...N_head
    gate_i,j^h = sigmoid(Linear(pair_i,j))                # gate_i,j^h ∈ ℝ^c
    q_i,j^h, k_i,j^h, v_i,j^h = Linear(pair_i,j, bias = False)   # q_i,j^h, k_i,j^h, v_i,j^h ∈ ℝ^c
    q_i,j^h = q_i,j^h / c^½
    k_i,j^h = k_i,j^h / N_atoms^½
    attn_i,j^h = softmax_j(sum_n((q_n,i^h)^T k_n,j^h) + bias_i,j^h)   # tied attention with bias; attn_i,j^h ∈ ℝ
    out_i,j^h = sum_k(attn_i,k^h v_j,k^h)
    out_i,j = flatten_h(out_i,j^h ⊙ gate_i,j^h)
    if is_row is True: out_i,j = out_j,i                  # flip rows and columns back
    return out_i,j
```

Algorithm 7. Dropout layer to zero out entire rows or columns from the pair representation

```
def TiedDropout(pair_i,j, p_drop = 0.15, is_row = True):
    mask_i ~ Bernoulli(1 - p_drop)                        # sample from Bernoulli distribution
    if is_row is True:
        pair_i,j = mask_i · pair_i,j / (1 - p_drop)       # drop row
    else:
        pair_i,j = mask_j · pair_i,j / (1 - p_drop)       # drop column
    return pair_i,j
```

Algorithm 8. Feed-forward layer

```
def FeedForward(x, c = 128, n = 2, p_drop = 0.10, normalize = True):
    if normalize is True: x = LayerNorm(x)
    x = Linear(x)                                         # x ∈ ℝ^cn (project up)
    x = Linear(Dropout(ReLU(x, p_drop)))                  # x ∈ ℝ^c (project down)
    return x
```

Algorithm 9. Triple product of three vectors

```
def TripleProduct(o, a, b, c):              # o,a,b,c ∈ ℝ^3
# input vectors are defined by 4 points; o is the initial point of the three vectors, a,b,c are endpoints
    v = (o − a) · ((o − b) × (o − c))       # v ∈ ℝ
    return v
```

Algorithm 10. Get gradients of bonded geometry w.r.t. atom coordinates

def GetGradients($\mathbf{X}_i$, bonds, bondlen, chirals, planars):

$\mathbf{X}_i$ = stopgrad($\mathbf{X}_i$)

$L^{bond} = \text{mean}_{i,j \in \text{bonds}} (\|\mathbf{X}_i - \mathbf{X}_j\| - \text{bondlen}_{(i,j)})^2$ # $L^{bond}, L^{chiral}, L^{planar} \in \mathbb{R}$

$L^{chiral} = \text{mean}_{o,i,j,k \in \text{chirals}} (\text{TripleProduct}(\mathbf{X}_o,\mathbf{X}_i,\mathbf{X}_j,\mathbf{X}_k) - 0.70710678)^2$

$L^{planar} = \text{mean}_{o,i,j,k \in \text{planars}} (\text{TripleProduct}(\mathbf{X}_o,\mathbf{X}_i,\mathbf{X}_j,\mathbf{X}_k))^2$

# we use pytorch automatic differentiation to calculate partial derivatives

$\mathbf{g}_i^{bond} = \partial L^{bond}/\partial \mathbf{X}_i$ # $\mathbf{g}_i^{bond}, \mathbf{g}_i^{chiral}, \mathbf{g}_i^{planar} \in \mathbb{R}^3$

$\mathbf{g}_i^{chiral} = \partial L^{chiral}/\partial \mathbf{X}_i$

$\mathbf{g}_i^{planar} = \partial L^{planar}/\partial \mathbf{X}_i$

$\mathbf{g}_i = \text{concat}(\mathbf{g}_i^{bond}, \mathbf{g}_i^{chiral}, \mathbf{g}_i^{planar})$ # $\mathbf{g}_i \in \mathbb{R}^{3\times3}$

return $\mathbf{g}_i$

Algorithm 11. Create *topk* neighbor graph

def MakeTopkGraph($\mathbf{X}_i$, separation, topk = 32):

edges = ∅

$D_{i,j} = \|\mathbf{X}_i - \mathbf{X}_j\|$

# for every node i, collect bonded and non-bonded neighbors

for all $i = 1,\ldots,N_{atoms}$:

  n_i^{bonded} = {topk/2 j's with smallest separation$_{i,j}$ and $i \neq j$}

  $n_i^{non\text{-}bonded}$ = {topk/2 j's with smallest $D_{i,j}$ which are not in n_i^{bonded}, $i \neq j$}

  $n_i = n_i^{bonded} \cup n_i^{non\text{-}bonded}$

  edges = edges ∪ {(i,j) **for** j **in** n_i}

*# create a graph**

$\boldsymbol{G}$ ← graph object with $V = 1,\ldots,N_{atoms}$ and E = edges

return $\boldsymbol{G}$

* Internally, we use DGL library to represent the graph in the SE3-Transformer module

Algorithm 12. Error prediction heads (plDDT, pDE, pDEV)

def PredictError($\mathbf{x}$, c=256, p_drop=0.0, bins=51):

$\mathbf{x}$ = LayerNorm($\mathbf{x}$)

$\mathbf{x}$ = Linear($\mathbf{x}$) # $\mathbf{x} \in \mathbb{R}^c$

$\mathbf{x}$ = Linear(Dropout(ReLU($\mathbf{x}$, p_{drop}))) # $\mathbf{x} \in \mathbb{R}^c$

$\boldsymbol{\varepsilon}$ = Linear(Dropout(ReLU($\mathbf{x}$, p_{drop}))) # $\boldsymbol{\varepsilon} \in \mathbb{R}^{bins}$

return $\boldsymbol{\varepsilon}$

Algorithm 13. All-atom FAPE loss

```
def GetFrame(a, b, c):
    u = b – a
    v = c – b
    e3 = (u – v) / ‖u – v‖
    e1 = v × u / ‖v × u‖
    e2 = e3 × e1
    R = concat(e1 , e2, e3)
    return R
```

Algorithm 14. All-atom FAPE loss

```
def FAPE({Xi}, {Yi}, S_frames, δ=1.0, cut=10.0):
    for all i = 1,...,N_atoms:
        for k,l,m in S_frames:
            R^X = GetFrame(Xk, Xl, Xm)
            R^Y = GetFrame(Yk, Yl, Ym)
            {Xi}^rot = R^X ({Xi} – Xl)
            {Yi}^rot = R^Y ({Yi} – Yl)
            {di} = {‖Xi – Yi‖}
    vi = 1/2{di}² if |{di}| < δ
    vi += δ*({di}-1/2 δ) if |{di}| >= δ
    vi = clamp(vi, cut)
    return meani(vi)
```

1.7. Analysis of potential data leakage for training on the CSD

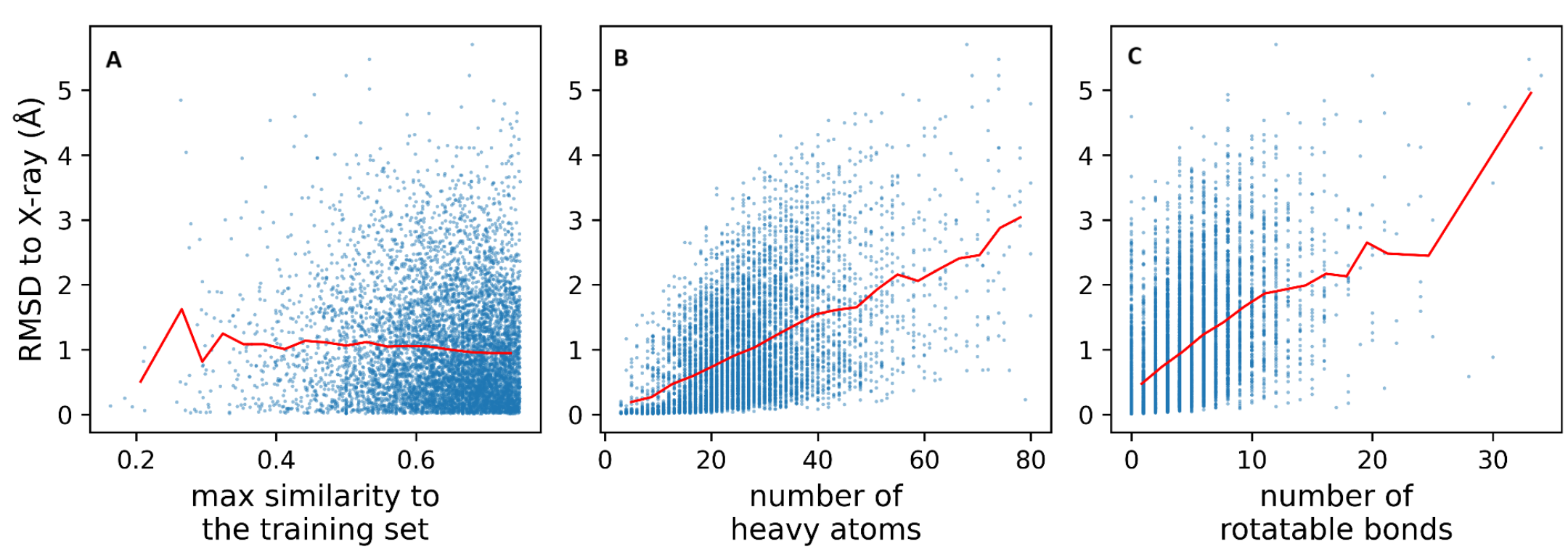

Figure S2. **Analysis of PLACER performance on the CSD test set**. **A)** Performance on the 7,116 test set examples is almost independent of the highest Tanimoto similarity to the test set. Factors like molecule size **(B)** and flexibility **(C)** on the other hand negatively affect PLACER performance. Trend lines are shown in red.

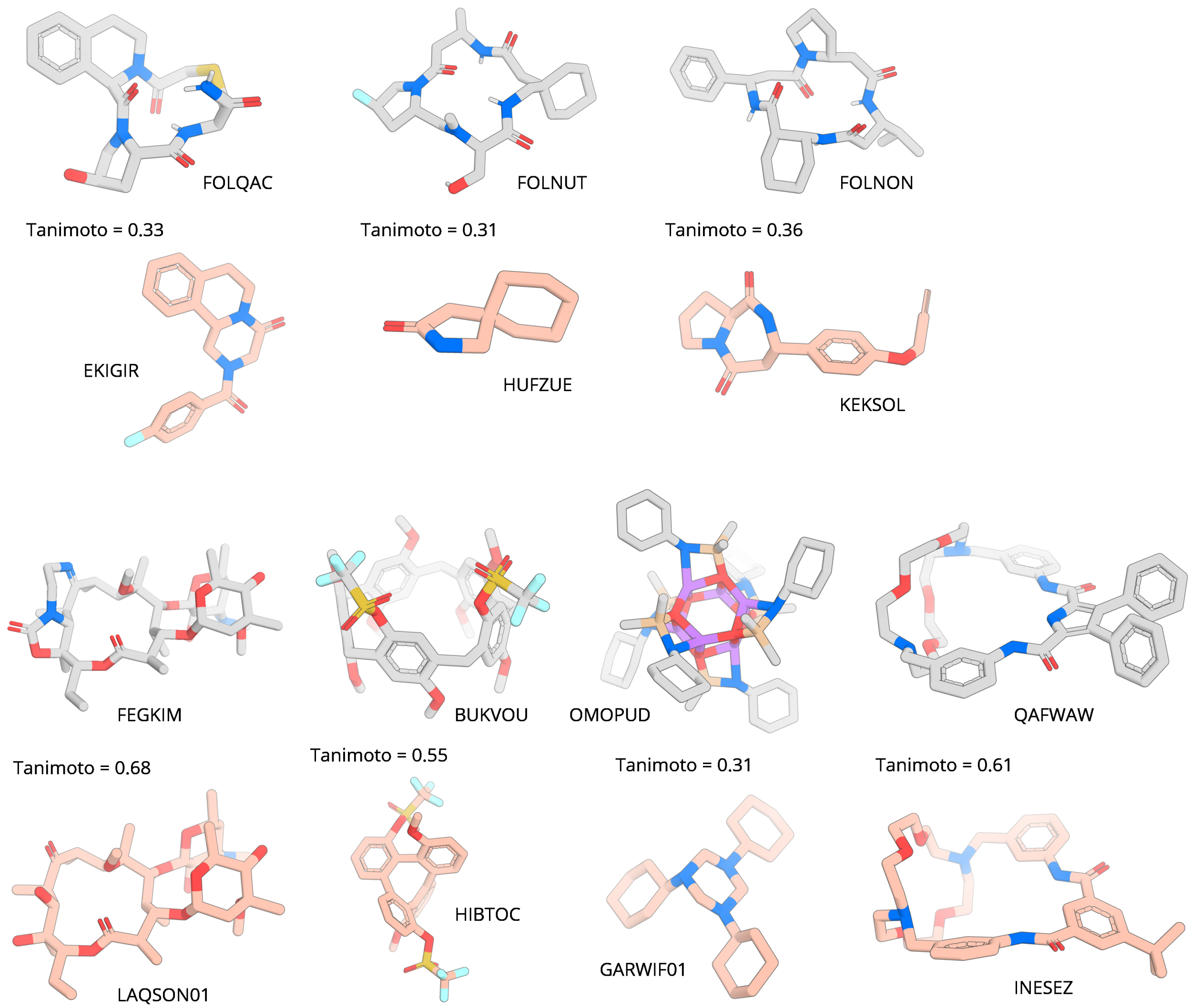

Figure S3. **Closest examples from the training set for the test molecules shown on Fig. 2D**. The former are shown in pale red while the latter are in grey. Respective CSD IDs are shown next to each molecule.

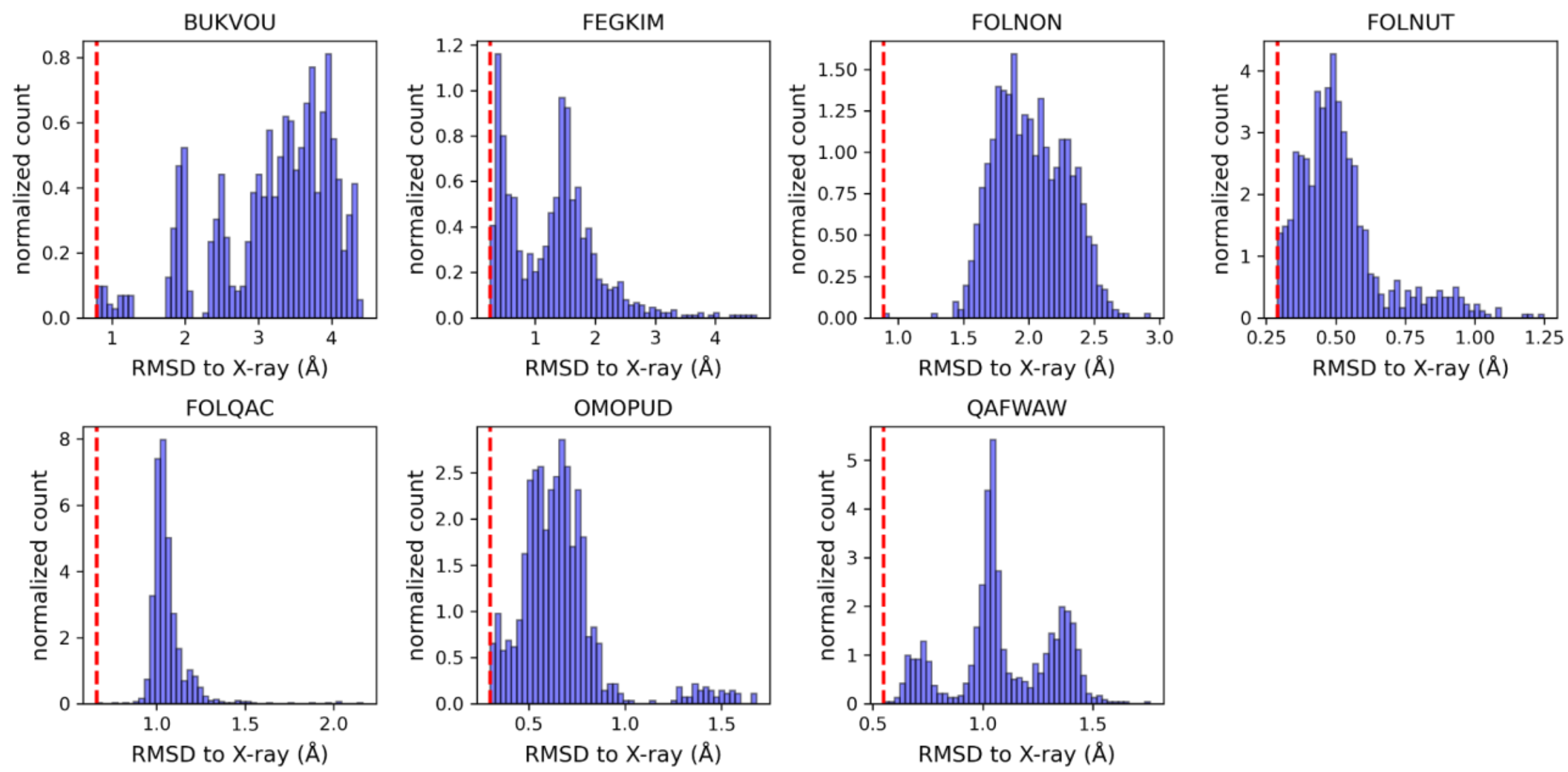

Figure S4. **RMSD distribution for all PLACER models generated for molecules shown on Fig. 2D.** Red dashed lines correspond to conformers with the lowest RMSD.

2. Astex non-native benchmark

To evaluate PLACER's performance in ligand docking tasks we applied it on the Astex non-native benchmark set, consisting of 65 target molecules, placed into their non-native protein structures, with a total of 1112 unique ligand-protein pairs.(2) This means, if protein X was crystallized with molecules M_1 (backbone X_1) and M_2 (backbone X_2) in the same pocket then in this benchmark we evaluate the docking of molecule M_1 into backbone X_2. The benchmark dataset was prepared by aligning backbone X_1 to X_2, and copying the coordinates of M_1 into backbone X_2, deleting molecule M_2 and any other ligands. PLACER was run on each protein-ligand pair, generating 1000 models. The performance of PLACER was evaluated by ranking the 1000 models based on three confidence metrics (pRMSD, pLDDT, pLDDT-pDE), and using RMSD of the highest confidence model as the result.

An example PLACER run command was as follows:

```
run_PLACER.py --ifile input.pdb -n 1000 --rerank prmsd
```

This will generate two outputs: `input.csv` containing the scores (pRMSD, plDDT, plDDT-pDE) and RMSD values of each model, ranked by pRMSD from lowest (best) to highest, and `input_models.pdb` containing the geometries of each of the 1000 models. The b-factor column in the PDB file represents the pRMSD metric for each atom. pRMSD value in the CSV file is averaged over all atoms of only the ligand.

We further explored the impact of applying physics-based minimization on the PLACER-produced models. For this, we used the minimization protocol available in GALigandDock(9) on all of the produced 1000 models. This protocol was applied using a Rosetta XML input (`eval.xml`) and the following run command:

```
$ROSETTA/main/source/bin/rosetta_scripts -s {input.pdb} @flags.hb -parser:protocol eval.xml -extra_res_fa {LIG.params} -constrain_relax_to_start_coords -gen_potential -overwrite -beta_cart -no_autogen_cart_improper -out:levels all:300 protocols.ligand_docking.GALigandDock:300 -out:prefix {outdir}/ -out:file:scorefile {outdir}/scores.sc -crystal_refine -mute core basic -constant_seed -jran 1234567
```

The produced models were ranked based on the computed dG metric, and the RMSD of the best model used as the final result.

eval.xml

```
<ROSETTASCRIPTS>
  <SCOREFXNS>
    <ScoreFunction name="genpot_soft" weights="beta_cart">
      <Reweight scoretype="fa_rep" weight="0.2"/>
    </ScoreFunction>
    <ScoreFunction name="genpot" weights="beta_cart">
      <Reweight scoretype="coordinate_constraint" weight="0.2"/>
    </ScoreFunction>
  </SCOREFXNS>
  <MOVERS>
    <GALigandDock name="dock" scorefxn="genpot_soft" scorefxn_relax="genpot"
                  runmode="eval" turnon_flexscs_at_relax="1" contact_distance="8.0" >
    </GALigandDock>
  </MOVERS>
  <PROTOCOLS>
    <Add mover="dock"/>
  </PROTOCOLS>
  <OUTPUT scorefxn="genpot"/>
</ROSETTASCRIPTS>
```

flags.hb

```
-beta
-score::hb_don_strength hbdon_GENERIC_SC:1.45
-score::hb_acc_strength hbacc_GENERIC_SP2SC:1.19
-score::hb_acc_strength hbacc_GENERIC_SP3SC:1.19
-score::hb_acc_strength hbacc_GENERIC_RINGSC:1.19
-no_autogen_cart_improper
```

Table S3. Astex non-native benchmark results with and without ligands at TC = 1.0 and TC ≥ 0.5 between the test set and the PDB training set.

	Full Astex non-native set		*Without* ligands with TC = 1.0*		*Without** ligands with* **TC ≥ 0.5**	
Metric	*< 1 Å* **accuracy**	*< 2 Å* **accuracy**	*< 1 Å* **accuracy**	*< 2 Å* **accuracy**	*< 1 Å* **accuracy**	*< 2 Å* **accuracy**
pRMSD	*41.8%*	*82.4%*	*40.8%*	*82.0%*	*39.2%*	*83.1%*
pLDDT-1D	*39.9%*	*78.6%*	*39.3%*	*78.3%*	*38.6%*	*80.2%*
pLDDT-2D	*37.6%*	*77.4%*	*36.5%*	*77.0%*	*34.5%*	*77.0%*

** 22 examples with TC = 1.0 ligands KAI, SWA and VIB were omitted from the benchmark set*

** 1296 examples with TC ≥ 0.5 ligands PH2, 675, AZM, A3M, SWA, NLA, IAD, BFL, IXM, STL, P16, 097, 3AR, GEO, THM, TQ3, NCT, VIB, AD3, KAI, SOX were omitted from the benchmark set.

Table S4. Tanimoto similarity coefficients for each of the ligands in the Astex non-native test set, compared against the PLACER PDB training set.

Astex ligand	Highest Tanimoto	Training ligand with highest Tanimoto		Astex ligand	Highest Tanimoto	Training ligand with highest Tanimoto
PH2	0.564	2PH		PM2	0.359	4PI
RQ3	0.471	E0O		SCT	0.371	AZZ
ID5	0.325	IFB		PFA	0.346	64L
JE2	0.476	E14		BYS	0.455	HF2
MTI	0.426	IRP		P16	0.608	96M
AO5	0.385	JOV		097	0.545	WR2
675	0.623	J4X		MOA	0.415	KP3
CIA	0.297	L8X		PFP	0.378	LIF
AZM	0.619	QOG		MC9	0.210	4D8
E4D	0.421	RAL		3AR	0.619	ARG
NDR	0.492	3WF		GIO	0.388	CHQ
A3M	0.710	WKM		GEO	0.593	CTN
BIT	0.303	YCJ		984	0.317	59U
**SWA**	**1.000**	**SWA**		LS1	0.382	QYZ
BDI	0.341	OHJ		TQD	0.262	W0S
STC	0.419	VWS		FR4	0.351	N0E
BSM	0.374	EH3		THM	0.667	AZZ
TNK	0.362	4WF		PH7	0.339	LJ5
CMU	0.245	I3V		PAF	0.422	TXI
NLA	0.541	NOA		BCZ	0.349	3XR
LI9	0.409	JQP		PVB	0.447	C1W
SKF	0.429	2CK		TQ3	0.744	JZM
IAD	0.556	E9M		NCT	0.575	HNK
BFL	0.564	9KL		**VIB**	**1.000**	**VIB**
IBA	0.313	4RB		CEL	0.467	4J8
FSN	0.384	UIR		905	0.330	J2Q
IXM	0.528	FEF		AD3	0.527	RFZ
BAU	0.351	4WF		CRZ	0.477	6H6
STL	0.543	R3S		**KAI**	**1.000**	**KAI**
HUP	0.226	OCH		CMB	0.488	IMA
BNE	0.245	JAN		SOX	0.629	PNN
BIR	0.477	BEY		198	0.389	4CX
ROF	0.338	W3B				

3. PoseBusters benchmark

To evaluate the ligand docking performance of PLACER against the protein-ligand co-folding methods AlphaFold3(10) and RoseTTAFold All-Atom(11), we used the PoseBusters benchmark set (as implemented in the 2023 preprint).(12) The dataset consists of 428 protein-ligand complexes, although at the time of writing this manuscript, one example (PDB id 7D6O) is no longer available in the Protein Data Bank. The predictions were performed based on the mmCIF files of the corresponding crystal structures. The target ligand was predicted with its coordinates randomly noised starting from the ground truth placement. Molecules present in the crystal structure that are known buffer components or crystallization aides were excluded from the prediction. The list of ligands that were avoided was obtained from the AlphaFold3 publication.(10) Other structurally relevant ligands and cofactors were kept approximately fixed during the prediction, if they were present in the 600-atom crop around the target ligand. The starting coordinates of those ligands were not noised prior to prediction - that way PLACER already starts with the true coordinates of those ligands, and through prediction usually their placement and conformation undergo minimal changes. For each example, 100 models were generated.

An example PLACER command that was used in PoseBusters benchmark:

```
python run_PLACER.py --cautious --exclude_common_ligands --rerank prmsd --
odir ./ -n 100 --ifile 5s8i.cif.gz --predict_ligand B-2LY-1501 --suffix B-
2LY-1501
```

A subset of 118 examples that are not present in the PLACER training and validation datasets were analyzed separately. The corresponding PDB id's are as follows:

```
5s8i, 6m2b, 6m73, 6xht, 6xum, 6yja, 6yqv, 6z4n, 6zpb, 6zr8, 7a9e, 7b94, 7bjj,
7cl8, 7ebg, 7ed2, 7fha, 7frx, 7ft9, 7jg0, 7jy3, 7kfo, 7kru, 7l03, 7l5f, 7lev,
7lt0, 7mae, 7mgt, 7nfb, 7nu0, 7oli, 7puv, 7qhl, 7qpp, 7qta, 7r59, 7rni, 7s45,
7s9h, 7sfo, 7sza, 7t1d, 7th4, 7tom, 7ts6, 7tsf, 7tuo, 7typ, 7uey, 7uj5, 7ujf,
7up3, 7ush, 7utw, 7uy4, 7uyb, 7v3n, 7vb8, 7vyj, 7wkl, 7wpw, 7wqq, 7wuy, 7xbv,
7xek, 7xi7, 7xjn, 7xpo, 7xqz, 7xrl, 7z2o, 7zcc, 7zdy, 7zhp, 7zl5, 7zoc, 7ztl,
7zu2, 7zxv, 8a1h, 8aem, 8aeu, 8ajx, 8auh, 8ay3, 8b8h, 8bn6, 8bom, 8bpl, 8bro,
8c3n, 8c5d, 8c5m, 8c7y, 8cgc, 8ci0, 8cnh, 8d19, 8dhg, 8dsc, 8eab, 8ex2, 8eye,
8f4j, 8f8e, 8fav, 8fln, 8flv, 8fo5, 8fv9, 8g0v, 8g43, 8g6p, 8gfd, 8h0m, 8ho0,
8slg
```

The ligand examples in the PoseBusters dataset were further analyzed for their similarity to ligands in the PLACER training set using the Tanimoto similarity coefficient. We found that of the 118 PoseBusters protein examples not present in the PLACER training and validation sets, 59 contained ligands that were relatively dissimilar, with TC <= 0.7 (Table S5 and Figure S5). Furthermore, 39 out of 118 ligands were still present in the PLACER training dataset, just as part of a different protein-ligand complex. Tanimoto Coefficients were calculated based on the Extended Connectivity Fingerprints (ECFP4)(13) using OpenBabel.(3)

Table S5. Tanimoto similarity coefficients (TC) for each of the ligands in the PoseBusters “not in training” n=118 subset, compared against the PLACER PDB training set.

Ligand	Highest TC	Training ligand with highest TC
76N	0.21	B0L
KUR	0.25	MQS
T78	0.25	5XV
E7K	0.26	JDD
N0R	0.28	H6M
7PP	0.29	JIY
KL9	0.30	NUM
D5P	0.31	WVY
I5F	0.31	ZEC
R3V	0.31	27P
NZ0	0.31	CQK
V6U	0.32	JFV
IAJ	0.33	T7A
98L	0.34	KTV
SMI	0.34	7P8
OK0	0.34	U7E
Q7B	0.35	PPT
60I	0.35	ILS
J0L	0.37	LWS
VUD	0.37	25J
F9F	0.37	GG8
VN2	0.37	5T2
R4W	0.38	ESA
IWE	0.38	U7E
Y7W	0.38	HW3
EZO	0.39	951
30L	0.39	KIU
TVW	0.39	1LQ
Q2Q	0.40	8T7
O88	0.41	4SX
VDX	0.42	HC3
ONJ	0.46	PFT
Y4U	0.47	AC0
80J	0.47	C
84Z	0.48	7VZ
QOZ	0.49	WGY
ZB9	0.49	MI1
GAR	0.50	FGR
KMI	0.51	0GD
T8E	0.51	9NB
3ZI	0.51	CHQ
ZHR	0.52	2F8
9YX	0.53	9QJ
TXV	0.53	1OO
STL	0.54	R3S
5Z6	0.54	156
R7E	0.54	MJO
2LY	0.56	Y34
OE3	0.59	QPZ
LVF	0.60	UOK
L9I	0.60	9X7
ZU6	0.64	EYA
IAC	0.66	3IO
4RI	0.66	CP7
X4I	0.66	J4S
TES	0.67	STR
5AD	0.67	QVR
XNG	0.68	11A
V2V	0.70	CTP
8HG	0.71	8OG
M0L	0.72	NUT
XJI	0.72	N6O
3D1	0.73	MDR
R53	0.73	NWK
MTA	0.73	NEA
URI	0.74	2TU
ANP	0.75	APR
YHT	0.75	PRX
ZD4	0.76	6AD
FWK	0.76	5FD
QU6	0.77	DXH
IQY	0.78	VP7
2BA	0.80	4BW
ADX	0.81	PPS
CA0	0.81	6R9
ATP	0.83	APR
A3P	0.84	PAP
ADP	0.84	ATR
XUS	0.90	TK7
FNR	1.00	FNR
8K2	1.00	8K2
4MB	1.00	4MB
0JO	1.00	0JO
GEN	1.00	GEN
DCL	1.00	DCL
ACO	1.00	ACO
DUI	1.00	Y70
FFO	1.00	FFO
H4B	1.00	H4B
DGL	1.00	GLU
82V	1.00	82V
NAI	1.00	NAI
AKG	1.00	AKG
CAQ	1.00	CAQ
F15	1.00	11A
APC	1.00	APC
NSD	1.00	NSD
UPG	1.00	UFM
FPF	1.00	FPF
OGA	1.00	OGA
6MJ	1.00	ARW
BCN	1.00	BCN
DHT	1.00	BDT
45D	1.00	45D
DLZ	1.00	DLZ
FUM	1.00	FUM
OJQ	1.00	OJQ
CP	1.00	CP
GTB	1.00	GTB
LMR	1.00	LMR
8EL	1.00	8EL
GSH	1.00	GSH
NCA	1.00	NCA
PHO	1.00	PHO
4Y5	1.00	4Y5
API	1.00	API
2EH	1.00	2EH
G5A	1.00	G5A

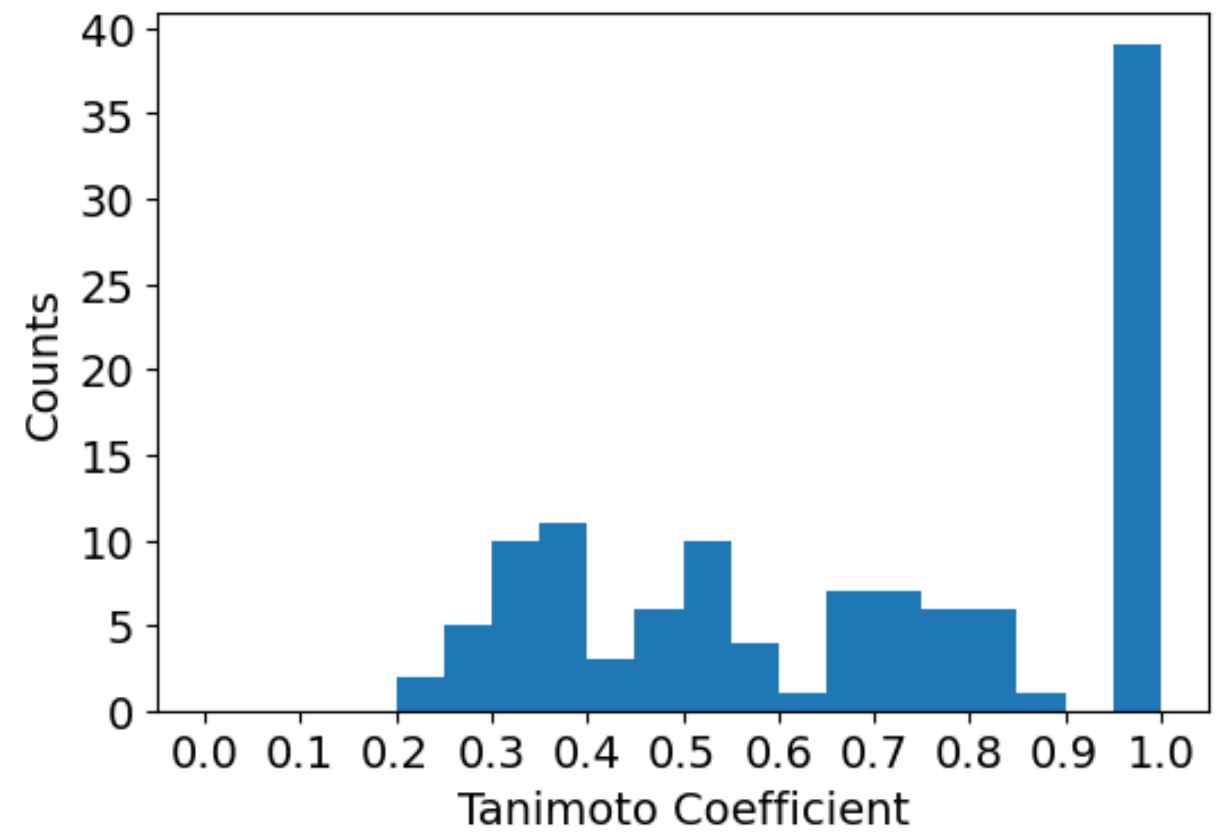

Figure S5. Distribution of Tanimoto similarity coefficient values shown in Table S5 between ligands in the PLACER training set, and ligands in the subset of PoseBusters dataset that was not part of the PLACER training and validation sets (based on PDB id's).

The models for each example were ranked by three different confidence metrics - pRMSD, plDDT from 1D and 2D tracks of the network. The RMSD between the ground truth and the top-ranking model was used as the representative result. As in the Astex benchmark, pRMSD was overall the most predictive of success (Figure S6), although interestingly in the n=118 subset, the 1D-track plDDT surpassed pRMSD in the higher accuracy <1 Å RMSD regime (57% vs 51% success rate, respectively). Furthermore, the subset of examples with ligands dissimilar to those used in PLACER training showed further drop in success rates in the high accuracy regime, but retained the success rates in the <2 Å RMSD regime. Although, with 59 examples in the latter subset, it might not be sufficiently large for drawing fully meaningful conclusions.

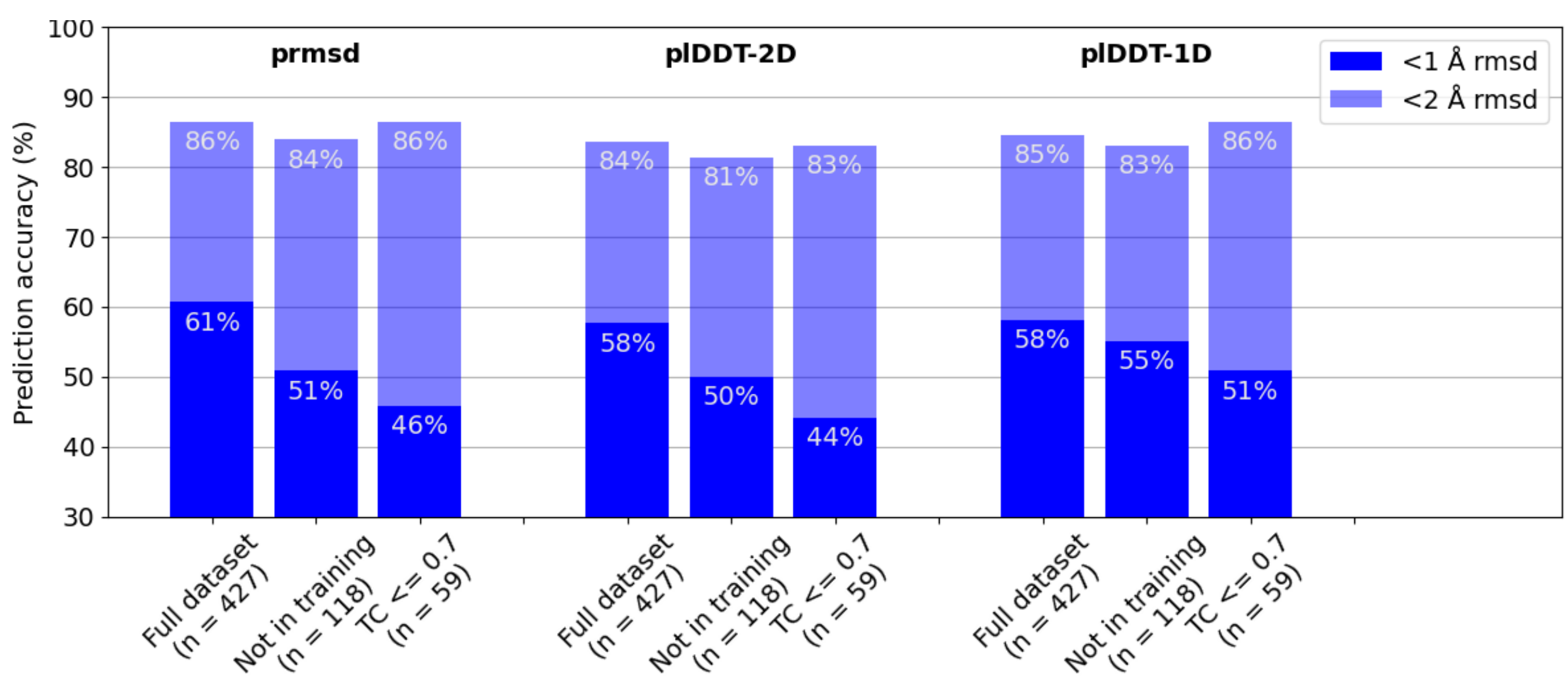

Figure S6. Success rates from predicting the PoseBusters dataset examples with PLACER, and ranking the models based on three different confidence metrics - pRMSD, 2D plDDT and 1D plDDT.

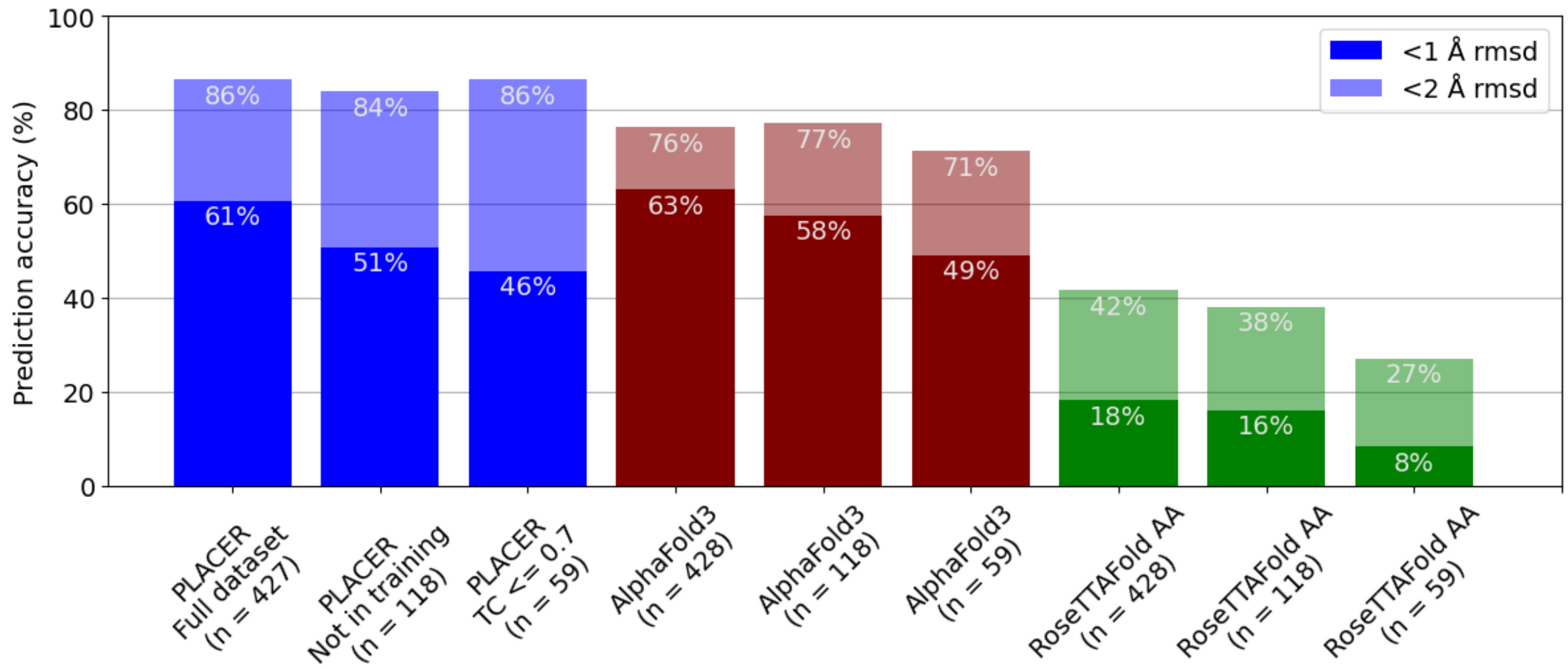

Figure S7. Comparison of PoseBusters success rates between PLACER (blue), AlphaFold3 (red) and RoseTTAFold All-Atom (green). PLACER results are based on ranking the models by pRMSD score. AlphaFold3 and RoseTTAFold All-Atom results were obtained from the respective publications. The n=118 subset corresponds to the examples not present in PLACER training and validation datasets. The n=59 subset corresponds to the examples with the ligand Tanimoto similarity coefficient <= 0.7 relative to the PLACER training set.

The PLACER outputs were further analyzed using the `bust` program from PoseBusters benchmark (https://github.com/maabuu/posebusters, downloaded on 20.08.2025). We assessed the quality of the best ranking dock (ranked by pRMSD) from each example in the n=118 subset with prediction RMSD < 2Å (99 models), with the results shown in Figure S8. The analysis revealed that PLACER overall produces chemically reasonable outputs, with very few failures with respect to fit with the rest of the protein and other cofactors. More failures arose in the molecular structure quality metrics, but this was largely limited by failures in the input sanitization step (parsing the molecule with RDKit), most likely caused by issues converting some of the PLACER outputs from the original PDB format to SDF format required by PoseBusters.

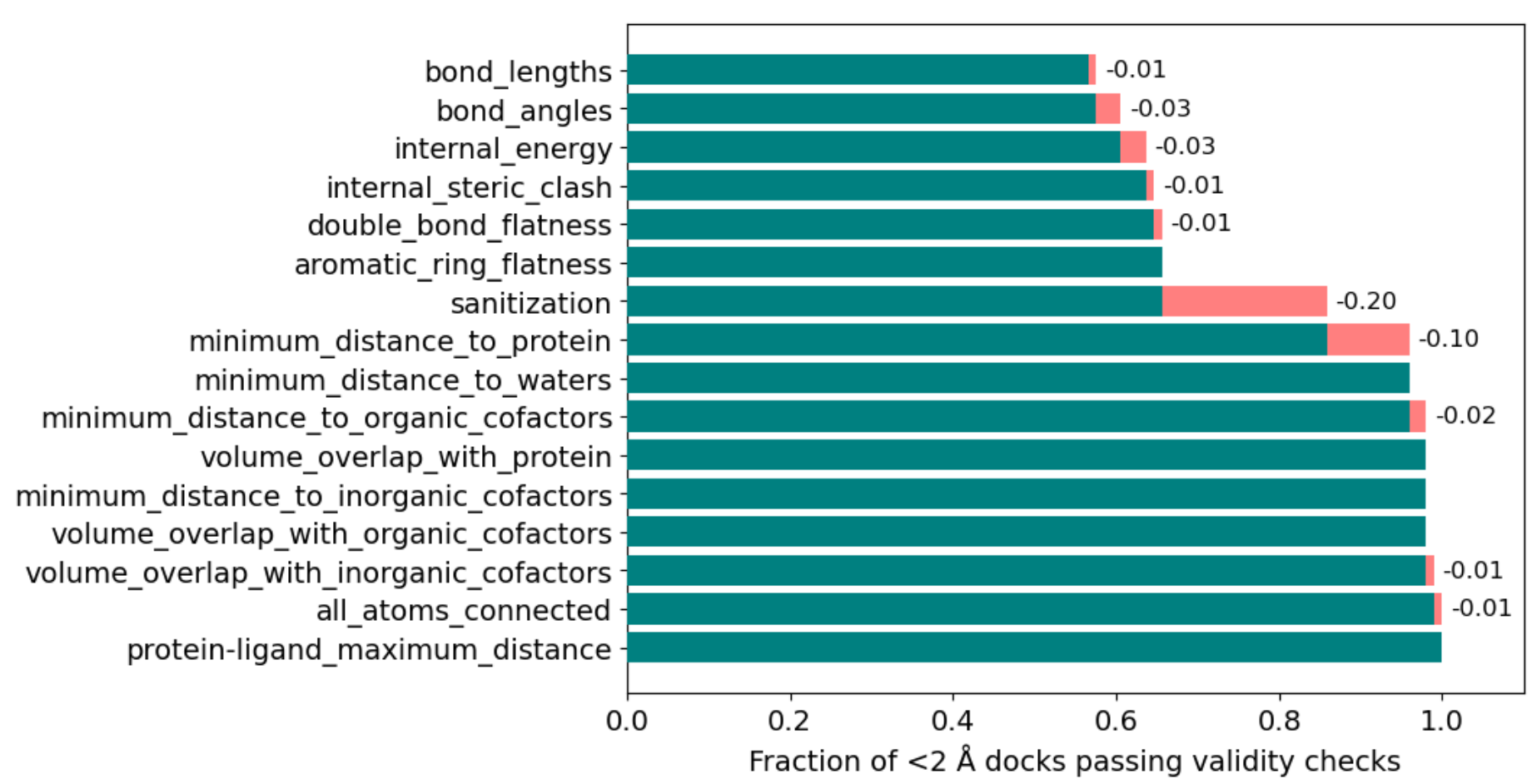

Figure S8. Results from running the PoseBusters filters on the best predicted structure from PLACER. Most violations occur in the “minimum distance to protein” metric.

4. Computational analysis of RA95 series of designed and evolved retroaldolases

To evaluate PLACER's ability to generate ensembles of enzyme active sites and use these models to discriminate between lower and higher activity variants we conducted an analysis on an existing set of enzymes with known activities. We chose the RA95-series of retroaldolase enzymes because there are high resolution crystal structures and detailed kinetic parameters of enzymatic activity for multiple variants along the evolutionary trajectory. The active sites of these enzymes center around nucleophilic lysine (K210 in RA95.0 and K83 in RA95.5 onwards) supported by an increasingly more sophisticated network of polar residues hydrogen bonding to the lysine and the substrate. In the reaction the lysine performs a nucleophilic attack at the carbonyl group of a beta-hydroxyketone substrate, which after shuffling of protons and departure of a water molecule undergoes a C-C bond cleavage reaction, yielding ultimately acetone and fluorescent 6-methoxy-2-naphthaldehyde (MeNA) products in case of the model methodol substrate (Fig. 4A). To perform the PLACER analysis on these active sites we focused on the covalently modified lysine at the different stages of the reaction - apo, methodol-carbinolamine (step 1), methodol-imine (step 2), acetone-imine (step 3), acetone-carbinolamine (step 4). We considered four different enantiomers of the methodol-conjugate; enantiomers with the lowest average predicted RMSD were used for analysis across the protein panel. To avoid irregularities of the crystal structures (missing loop densities, alternative atom placements), we collected sequences of the RA95 retroaldolases and predicted their structures using ColabFold following relaxation of the best ranking model with AmberRelax(14). Resulting models had Cα RMSDs between 0.15 and 0.47Å to corresponding crystal structures and were used for PLACER computations. We generated 50 samples with PLACER at each step and found that both the RMSD and the predicted RMSD (pRMSD) of the conjugated methodol correlated well with the experimentally determined catalytic activity of these variants (Fig. S8 and 4B). It is noteworthy that the trend is stronger when focusing on the methodol-conjugated lysine, and non-existent for acetone-conjugated lysine, highlighting PLACER's ability to consider the impact of the true substrate.

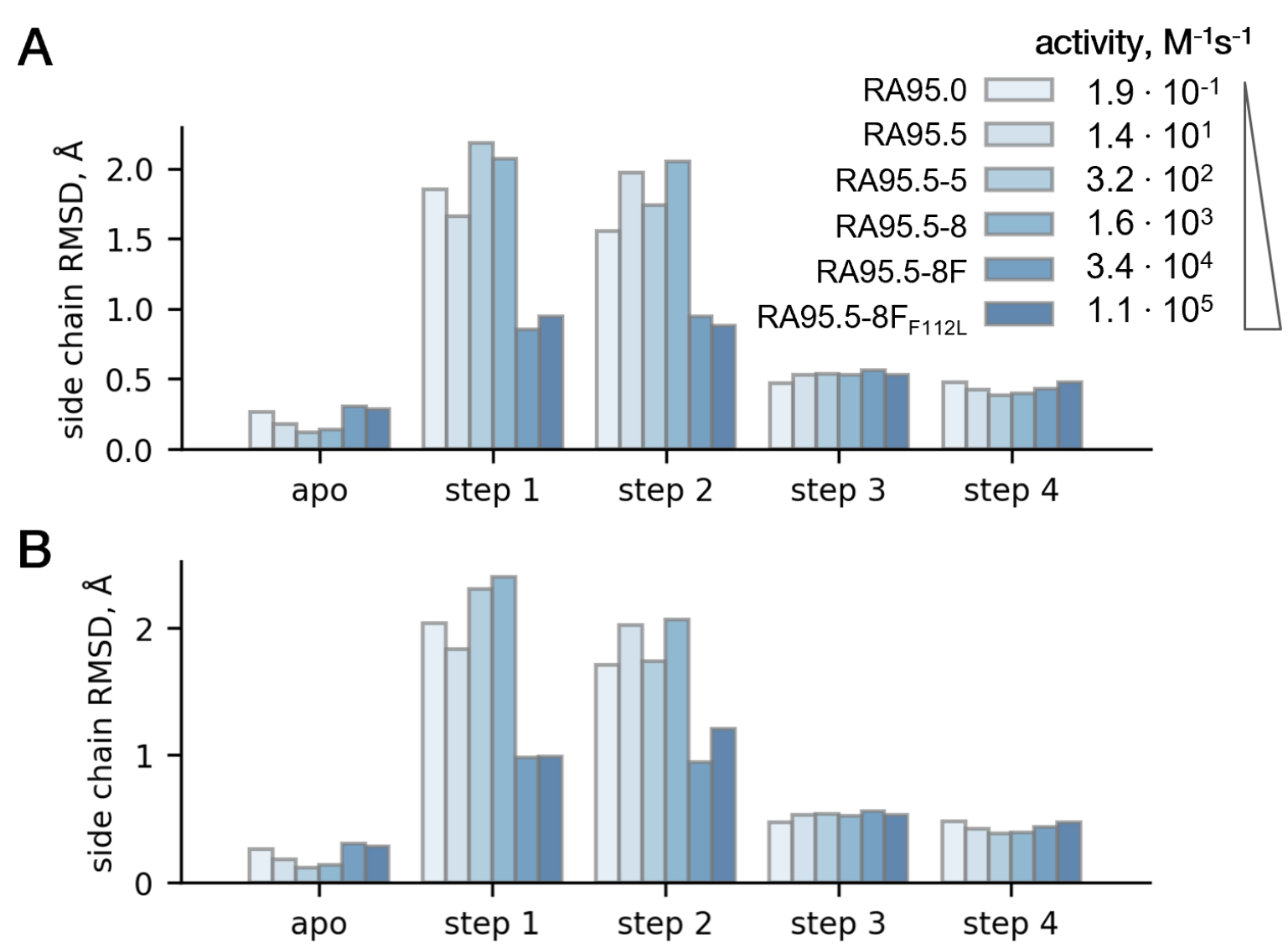

Figure S9. Five intermediate states of the RA reaction with methodol were modeled by PLACER for the RA95 series of designs to probe preorganization of the active site lysine and its modifications. Shown is the analysis based on the RMSD of the free lysine (*apo*) or conjugated lysine atoms except N, Cα, C, O atoms. **A)** Enantiomer with lowest RMSD is used for step 1 and step 2 in the top panel. **B)** RMSDs for *R,R*-enantiomer are in the lower panel.

5. Computational de novo design of retroaldolases in NTF2 fold

We explored the ability of PLACER to aid in the selection of higher activity de novo designed enzymes, using the retro-aldol reaction as an example.

A short summary of the workflow for evaluating PLACER in the context of enzyme design:

1. Design methodology development using de novo generated NTF2 scaffolds
2. Experimental identification of most viable active site configuration
3. Development of PLACER "selection model" based on the data from step 2
4. IVTT based increased throughput retroaldolase activity testing assay
5. Individual purification and activity testing of selected designs
6. In depth PLACER analysis of the experimentally tested designs

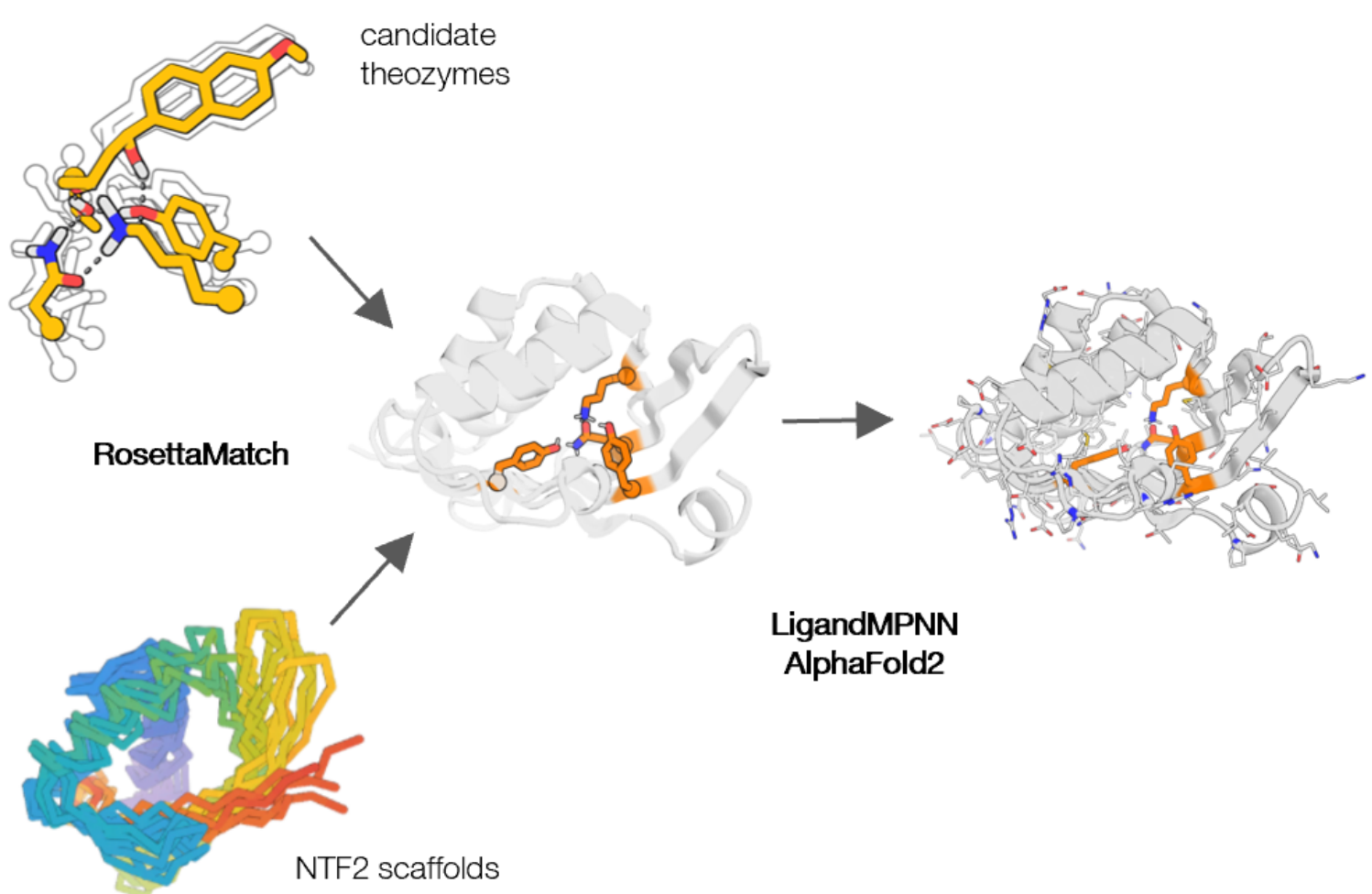

Figure S10. Overview of the design workflow.

Successful design process requires to address following challenges: generation of large number of designable backbone variations of NTF2 fold, identification of potential locations where catalytic quartet can be installed and finally coming up with selection criteria to downsample number of designs for experimental testing. To address the first challenge we used RoseTTAFold joint inpainting (RFjoint2)(15) to generate complete NTF2 scaffolds by randomly retaining 3 or 5 contiguous residues in major strands and helices of the randomly picked "seed" NTF2 structure, and allowing RFjoint2 to fill in missing pieces of the backbone. For each generated scaffold TM score relative to the "seed" structure was computed and 10000 scaffolds with the score greater than 0.62, which ensured NTF2-like topology, were selected for the next stages.

To install the catalytic quartet we used a two step procedure. We first used a modified XML-Matcher protocol(16) to determine what positions in the NTF2 fold might be broadly compatible with geometric requirements of the four residues interacting with the ligand and forming a hydrogen bond network. Instead of exhaustive enumeration of all possible spatial arrangements consistent with desired connectivity, we implemented a guided Monte-Carlo docking procedure to bring disjointed randomly placed residues together to form a theozyme as illustrated in Figure S11. By repeating docking trajectories of the disembodied sidechain functional groups with each of the 4 possible stereoisomers of the carbinolamine intermediate, and removing outputs that did not have desired connectivity, we generated 4 ensembles of several thousands structures each. The number of successful trajectories varied substantially between stereoisomers (*R,R* – 4352 out of 5000, *R,S* – 2177, *S,R* – 4373, *S,S* – 2910), suggesting structural

preferences of the catalytic quartet. After grouping generated structures by similarity of *nucleophile* ("nuc") and *shuttle* ("sht") positions (183 total clusters generated, split between *R,R* – 20, *R,S* – 23, *S,R* – 77, *S,S* – 63), geometric parameters describing rigid body transformations between reference atoms of the ligand and reference atoms of the protein side chains of cluster members were extracted and written into XML-formatted constraint files. Additional theozyme diversity was incorporated by allowing the *bridge* ("brd") residue to be either Asn or Gln, and *support* ("sup") to be Tyr, Ser or Thr, resulting in 6 possible theozyme compositions. This approach is not as thorough in exploring all possible ways to arrange theozyme components in space as it would be with HBNetGen(16), but more rapidly generates diversified theozyme coordinates and enables exploration of alternative site descriptions. This more rapid feedback was necessary as initial attempts to match catalytic quartet theozymes in native scaffolds resulted in a large fraction of the output having theozyme residues placed on the unstructured looped regions of the scaffolds. To enrich sampled theozyme geometries with those compatible with catalytic residues projecting from ordered secondary structure elements, we repeated the docking protocol with theozyme residues in tripeptide segments of helical or extended backbones; this resulted in more matches in regular secondary structure elements. In total 6*183=1098 individual constraint files were generated and used for matching.

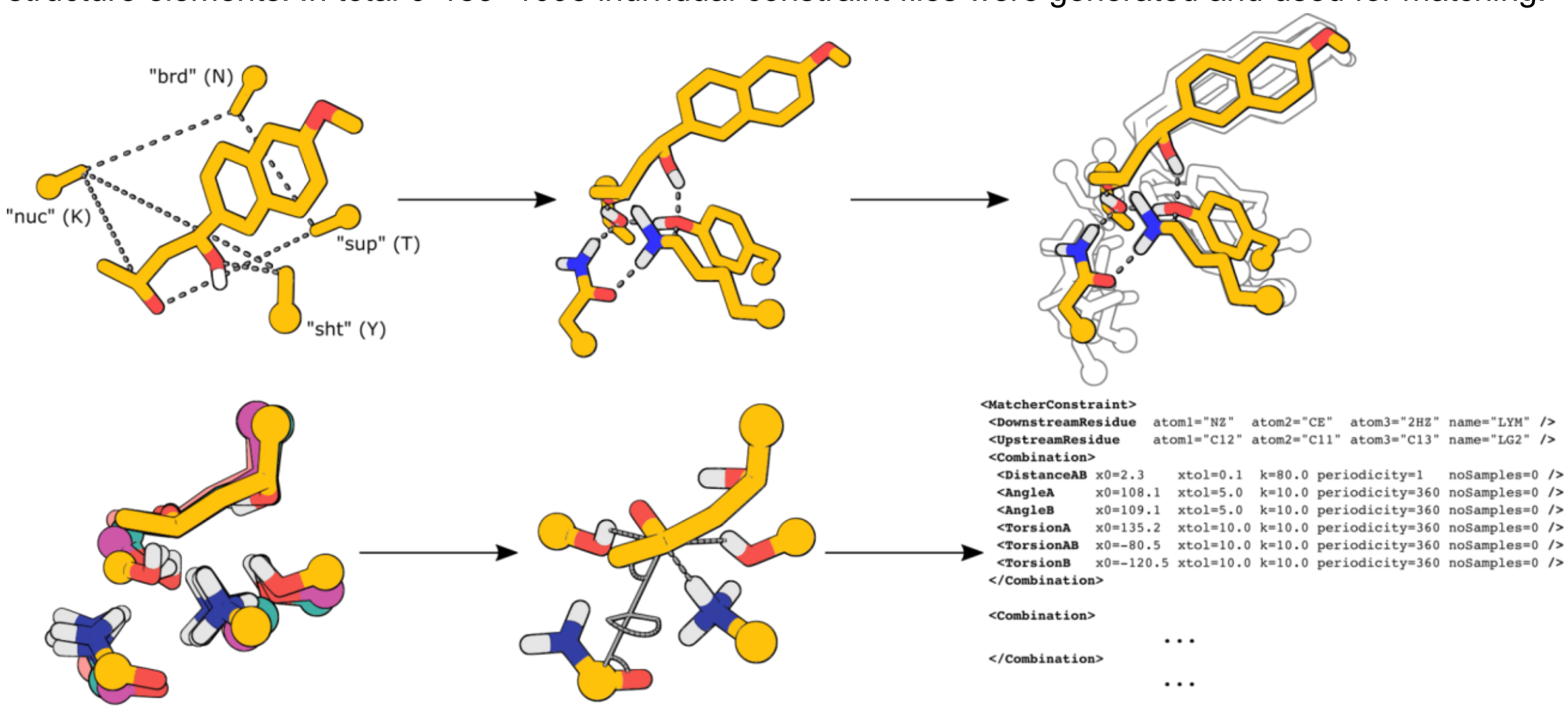

Figure S11. Preparation of theozyme geometries with a guided Monte-Carlo docking procedure and converting them into xmlMatcher formatted constraints. (top panel) each docking trajectory is initialized form theozyme components randomly oriented in space and brought into desired geometry by cycles of rigid body perturbations and restrain guided minimization with Rosetta; (bottom panel) rigid body orientation geometries of chemical motifs are extracted from ensemble and written into xmlMatcher formatted text file

Since the protocol is computationally intensive, we performed the computation for 500 out of 10000 randomly selected NTF2 backbones. We identified several combinations of positions and theozyme residue types that frequently produced reasonably good approximations of the desired geometry in multiple backbones. Following this preliminary stage, we used structurally equivalent positions in all 10000 scaffolds to install theozyme residues and design the rest of the positions in the backbone using structure conditioned sequence design in the presence of the substrate (LigandMPNN)(17) to generate multiple sequences for each backbone. AlphaFold2(7) was used to predict the structure of each sequence (in single sequence prediction mode, using model 4 ptm and 3 recycles), and models with lDDT higher than 85 created a pool of designs subjected to various *in silico* selection strategies.

5.1. Identification of focus theozyme configuration

The space of possible theozyme configurations (catalytic residue placement and amino acid identities) in any given protein topology is finite and limited by the geometry of the interacting theozyme side chains. However, not every configuration, while consistent from geometric perspective, is going to be inherently catalytically competent. Therefore, to be able to focus on assessing the role PLACER in the design workflow, we needed to establish conditions such that a reasonable number of active designs could be expected in the batch of experimentally tested variants.

We ran a preliminary experiment where a batch of 90 designs was split between three most promising theozyme configurations (Table S6) identified using XML-Matcher protocol (34, 35, and 21 designs for configurations 1, 2 and 3 respectively).

Table S6. Tested configurations of and placements of retro-aldolase theozymes

Configuration	"nuc"	"brd"	"sup"	"sht"
1	K80	N78	S91	Y108
2	K62	Q78	Y104	Y89
3	K106	Q104	Y13	Y52

Selection criteria for individual designs were based on how well side chains of the theozyme residues recapitulated desired geometry in the AF2 predicted structures with pLDDT > 85 and did not include PLACER metrics. Results of activity testing using IVTT protocol (Figure S12) suggested that one of the theozyme configurations may be more designable using our computational pipeline.

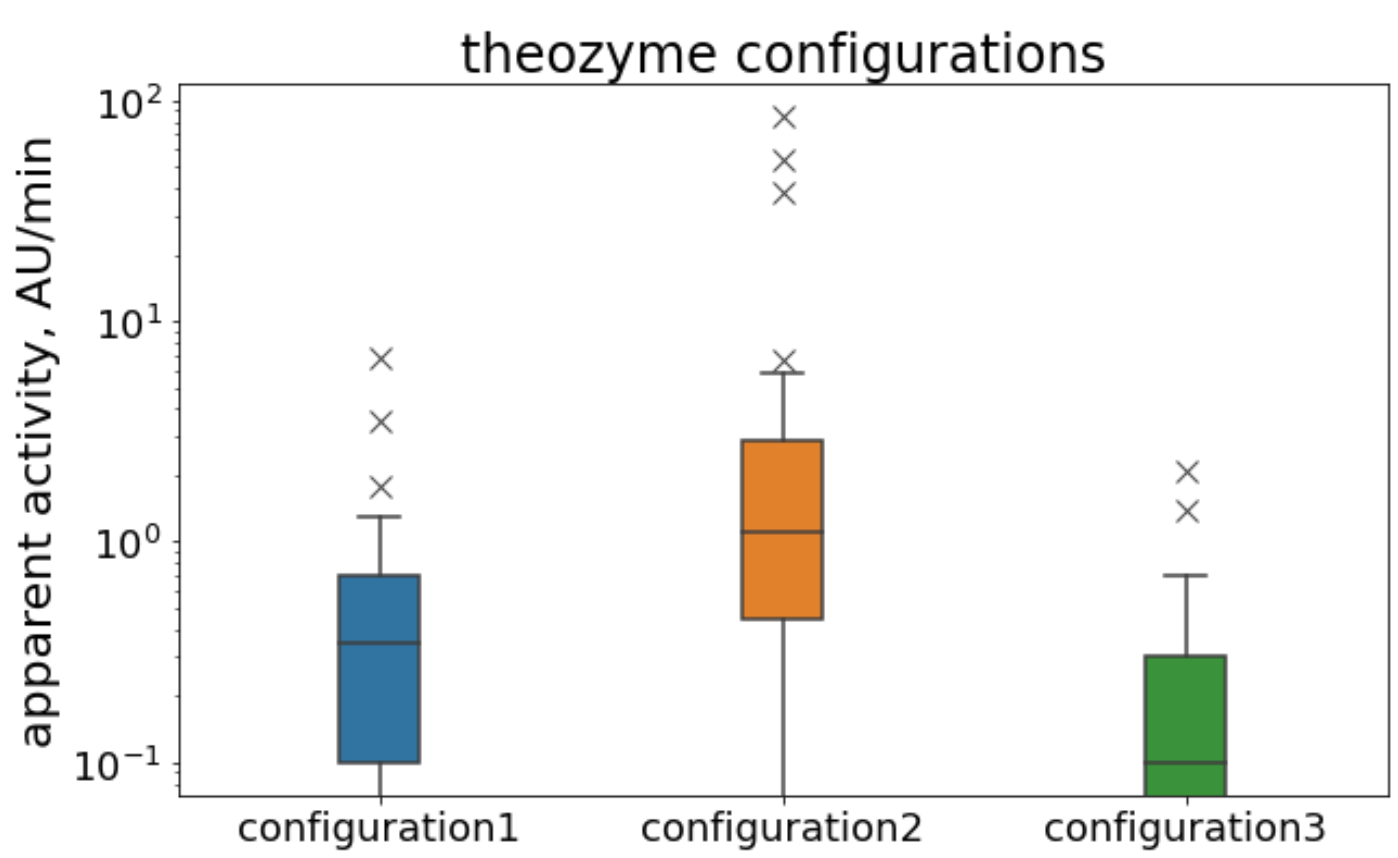

Figure S12. Distribution of apparent activities for designs with various theozyme configurations. Configuration 2 appears to be more compatible with underlying requirements of retro-aldolase active site geometry and was used to generate a pool of designs for testing PLACER selection strategy.

As a result of this experiment we decided to use configuration 2 to generate the common pool of designs used to test computational selection strategies.

5.2. Application of PLACER for design selection

To test various downsampling selection strategies we generated a common pool of designs by installing theozyme configuration 2 into 10000 NTF2-like scaffolds (368 scaffolds in which at least one component of the theozyme could not be mapped into structurally equivalent environment were omitted from design calculations). For each of the 9632 scaffolds 8 sequences were generated with LigandMPNN, keeping residue type of the theozyme positions fixed, and for each sequence its structure was predicted with AlphaFold2. Structures with pLDDT ≥ 85 (17180 in total) formed the pool of designs used to select variants for experimental testing.

(i) First, “naive” strategy relies on the hypothesis that AlphaFold2 predicted positions of the theozyme side chains in the *apo* form of the design correlate with actual conformations of these residues both in *apo* and *holo* form of the design. From the pool of designs we would select ones where the AlphaFold2-predicted *apo* structures have the theozyme residues participating in required hydrogen bonds.

(ii) Second strategy relied on using PLACER as the design evaluation tool. For each design, we would run PLACER 50 times, creating an ensemble of conformations. A set of six metrics, such as uncertainties in the positions of the polar atoms, and average distances between hydrogen bonding atoms of the theozyme residues were computed across ensemble members.

Base selection strategy, dubbed “naive”, used a set of interatomic distances between polar atoms of the theozyme side chains “nuc(NZ)”-“brd(OE1)”, “sup(OH)”-”brd(NE2)”, “nuc(NZ)”-”sht(OH)” in the predicted structure as a proxy for formation of the hydrogen bonding network in the theozyme in the absence of the ligand. This was complemented by RMSD of the theozyme side chain heavy atoms between AlphaFold2 predicted structure and a structure of the pre-LigandMPNN design with theozyme geometry optimized to reflect preconceived vision of transition state stabilization by the protein. Numerical values for the distances and RMSD cutoffs were adjusted to result in 90 designs passing the selection (RMSD < 1.73 Å, “nuc(NZ)”-“brd(OE1)”, “sup(OH)”-”brd(NE2)” and “nuc(NZ)”-”sht(OH)” between 2.0 and 3.3 Å). DNA sequences encoding for these designs were obtained as IDT eBlocks™ and their activity tested following the IVTT protocol.

Selection strategy, dubbed “PLACER”, utilizing PLACER to assist in picking the most promising designs from the same pool of 17810 variants relies on a set of geometrical and uncertainty metrics from generating an ensemble of 50 structures with PLACER, predicting interactions between the ligand and the active site residues in the context of AlphaFold2 predicted backbones. The obtained metrics included distances between polar atoms of the theozyme side chains and uncertainty (*pRMSD*) in polar atoms positions computed over the ensemble of 50 models. To identify a smaller subset of PLACER metrics we trained a simple logistic regression model to classify designs as active or inactive on a set of designs characterized in the preliminary experiment performed to identify focus theozyme configuration.

5.3. IVTT based retroaldolase activity testing assay

We developed a protocol for medium throughput semi-quantitative retro-aldolase activity testing of the designs using in vitro transcription translation system (IVTT). We obtained linear duplex DNA, encoding protein of interest, flanked by appropriately spaced T7 promoter and T7 terminator sequences as eBlocks™ gene fragments from IDT, and by mixing it directly with 2 μL components of PurExpress IVTT system produced enough protein to screen for active variants. Genes of several control proteins with a range of activities previously determined in purified form were included with each batch of tested designs to assist in ranking activity of the new variants, and help to compare experiments run under different experimental conditions. It should be noted that in this format of the experiment, concentration of the soluble protein remains unknown and therefore activity level should be treated as apparent activity defined as a product of the amount of soluble protein and intrinsic activity of each variant. It is possible to make assay more quantitative by fusing protein of interest to fluorescent protein reporter at the expense of decreased amount of retro-aldolase catalyst synthesized. Typically, a batch of 90 designs and 6 controls were used to evaluate performance of a particular computational workflow.

5.4. Characterization of individual variants

To get more accurate and quantitative data describing catalytic activities of the designs and validate IVTT based assay, we cloned, expressed in Escherichia coli, purified and determined Michaelis-Menten parameters for twelve designs with highest apparent activity from "naive" or "PLACER" group (24 proteins in total). Additionally, we purified and tested under similar experimental conditions three previously characterized proteins possessing retro-aldolase activity. This group of control proteins consists of published RA95.5-8F(18), RA110-4.6(19), and RA**β**b-16.2(20) retro-aldolases computationally designed and subjected to various extent of directed evolution optimization, leading to significant improvements in catalytic proficiency.

Under generic unoptimized conditions, soluble expression levels in *E. coli* varied almost 100-fold among 24 proteins, indicating the possibility that when uncorrected for concentration, IVTT determined activities may be unreliable in recovering high activity variants. However, activity of the designs expressed in terms of k_{cat}/K_M correlate qualitatively (R^2 ~0.4) with apparent activity measured in IVTT experiment, suggesting that findings on the smaller subset of purified proteins can be, to certain extent, extrapolated onto the larger subset of proteins characterized in IVTT format. Moreover, the highest activity variants in the "PLACER" group found in IVTT experiment are indeed the highest activity variants by their k_{cat}/K_M values, except two variants in IVTT assay that appeared to have activity level beyond the dynamic range of the assay, but in purified form demonstrated modest levels of activity. Mean k_{cat}/K_M values in "naive", "PLACER", and "control" groups are 250, 2300, and 80,000 M^{-1} min^{-1} respectively.

Furthermore, results obtained with purified proteins confirm the initial observation that designs cnRA-34 and cnRA-50 in the "PLACER" group, with k_{cat}/K_M values of 6900 and 11,000 M^{-1} min^{-1} respectively, are the most efficient computationally designed catalysts that were not subjected to additional experimental optimization, and being similar to the activity of the RA**β**b-16.2 (12,000 M^{-1} min^{-1}, 5 rounds of evolution) and only surpassed by RA95.5-8F (240,000 M^{-1} min^{-1}, 18 rounds of evolution; it should be noted that published estimates for k_{cat}/K_M of this enzyme are 5-10 times higher most likely due to phosphate based buffer used in this study, which was shown to have strong inhibitory effect on retro-aldolases built on 1a53 scaffold(21)) (Figure S13).

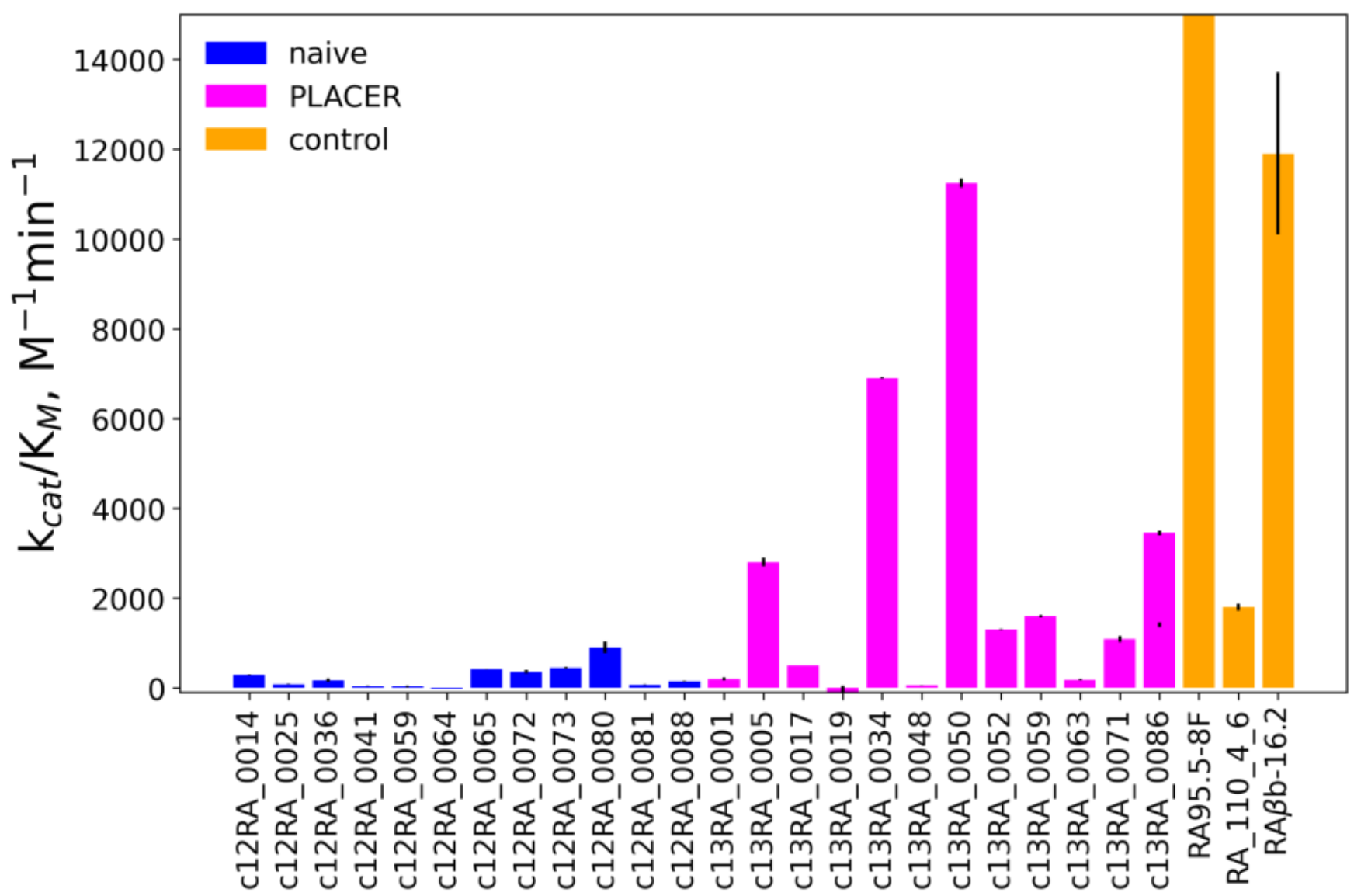

Figure S13. Values of k_{cat}/K_M for top 12 designs from "naive" and "PLACER" group.

5.5. In depth PLACER analysis of the experimentally tested designs

Obtaining a substantial number of designs with detectable level of activity allowed us to perform analysis analogous to that of the RA95 series of retroaldolases (Fig. 4B and Figure S9) on all experimentally characterized (either in IVTT or in purified protein format) de novo NTF2 designs (548 total), and see the level of discrimination between active and apparently inactive variants according to PLACER ensembles. For each design we generated 50 samples for each enantiomer of methodol-carbinolamine (step 1) or methodol-imine (step 2) intermediates. Due to an opening created by PLACER pose cropping (<600 atom crop) of NTF2 topology, some models in the PLACER ensemble have an incomplete representation of the pocket and allow the buried catalytic residues to "escape" the protein active site, artificially increasing values of ensemble RMSD. To mitigate this effect, we removed from the analysis members of the ensemble deviating more than two standard deviations from the ensemble centroid. We found that both the RMSD and the pRMSD of methodol atoms in conjugated lysine correlated well with the experimentally determined catalytic activity of these variants (Fig. S14 and 4F). It should be noted that none-zero fraction of designs may be falsely labeled as inactive in IVTT assay due to errors in coding DNA eBlock synthesis or low soluble expression in a non-cellular environment, and may contribute to distribution overlap, making discrimination less clear.

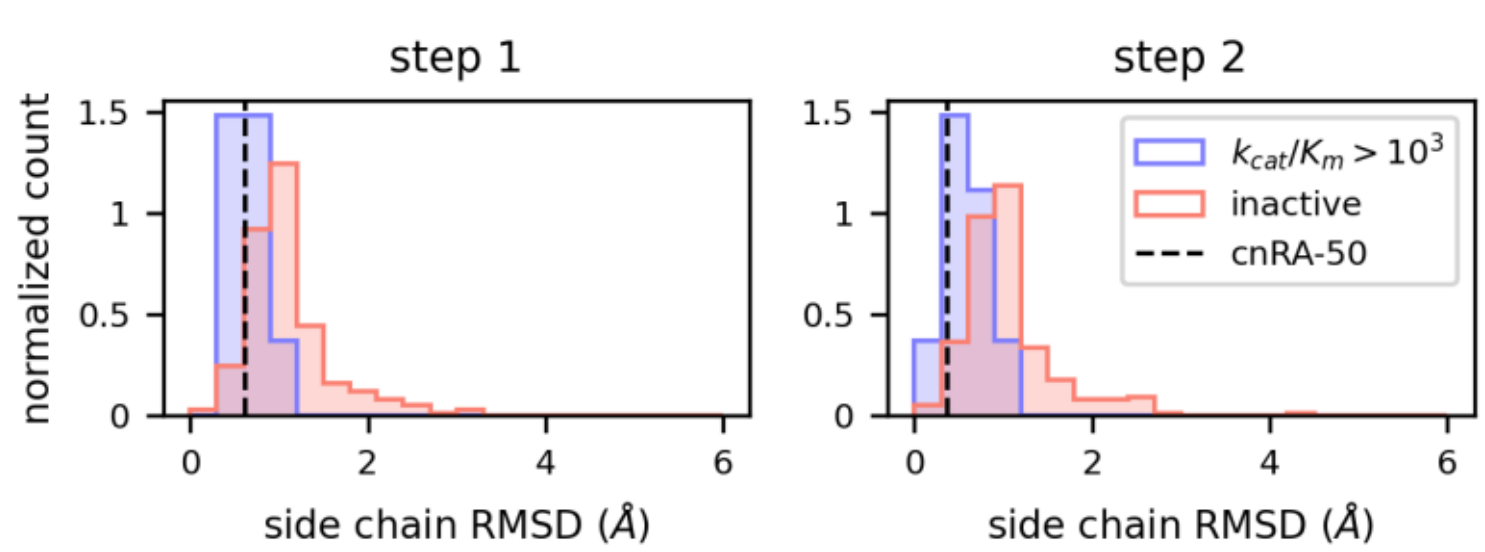

Figure S14. PLACER active site pre-organization ensemble metrics (average RMSD of conjugated lysine atoms except N, Cα, C, O to ensemble centroid positions) in steps 1 and 2 enrich for selecting more active de novo designed retro-aldolase enzymes.

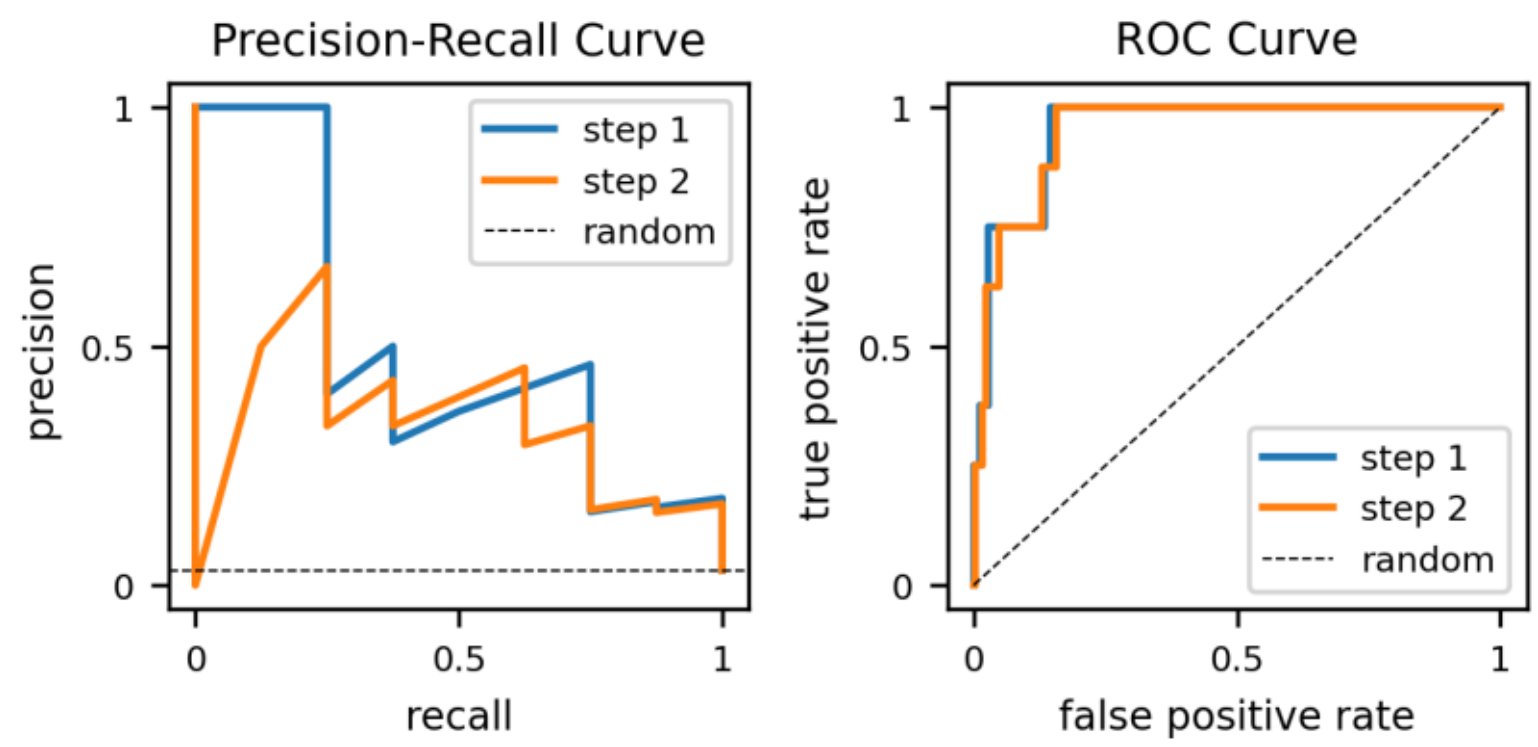

Figure S15. Separation between pRMSD distributions for active and inactive designs from Fig. 4F shown in terms of PR and ROC curves.

6. References

1. H. M. Berman, *et al.*, The Protein Data Bank. *Nucleic Acids Res.* **28**, 235–242 (2000).
2. M. L. Verdonk, P. N. Mortenson, R. J. Hall, M. J. Hartshorn, C. W. Murray, Protein-ligand docking against non-native protein conformers. *J. Chem. Inf. Model.* **48**, 2214–2225 (2008).
3. N. M. O'Boyle, *et al.*, Open Babel: An open chemical toolbox. *J. Cheminform.* **3**, 33 (2011).
4. J. D. Westbrook, *et al.*, The chemical component dictionary: complete descriptions of constituent molecules in experimentally determined 3D macromolecules in the Protein Data Bank. *Bioinformatics* **31**, 1274–1278 (2015).
5. J. Dauparas, *et al.*, Robust deep learning-based protein sequence design using ProteinMPNN. *Science* **378**, 49–56 (2022).
6. C. R. Groom, I. J. Bruno, M. P. Lightfoot, S. C. Ward, The Cambridge Structural Database. *Acta Crystallogr B Struct Sci Cryst Eng Mater* **72**, 171–179 (2016).
7. J. Jumper, *et al.*, Highly accurate protein structure prediction with AlphaFold. *Nature* **596**, 583–589 (2021).
8. N. Hiranuma, *et al.*, Improved protein structure refinement guided by deep learning based accuracy estimation. *Nat. Commun.* **12**, 1340 (2021).
9. H. Park, G. Zhou, M. Baek, D. Baker, F. DiMaio, Force Field Optimization Guided by Small Molecule Crystal Lattice Data Enables Consistent Sub-Angstrom Protein-Ligand Docking. *J. Chem. Theory Comput.* **17**, 2000–2010 (2021).
10. J. Abramson, *et al.*, Accurate structure prediction of biomolecular interactions with AlphaFold 3. *Nature* **630**, 493–500 (2024).
11. R. Krishna, *et al.*, Generalized biomolecular modeling and design with RoseTTAFold All-Atom. *Science* **384**, eadl2528 (2024).
12. M. Buttenschoen, G. M. Morris, C. M. Deane, PoseBusters: AI-based docking methods fail to generate physically valid poses or generalise to novel sequences. *Chem. Sci.* **15**, 3130–3139 (2024).
13. D. Rogers, M. Hahn, Extended-connectivity fingerprints. *J. Chem. Inf. Model.* **50**, 742–754 (2010).
14. G. Kim, *et al.*, Easy and accurate protein structure prediction using ColabFold. *Research Square* (2023).
15. D. E. Kim, *et al.*, Parametrically guided design of beta barrels and transmembrane nanopores using deep learning. *bioRxivorg* 2024.07.22.604663 (2024).
16. B. D. Weitzner, Y. Kipnis, A. G. Daniel, D. Hilvert, D. Baker, A computational method for design of connected catalytic networks in proteins. *Protein Sci.* **28**, 2036–2041 (2019).
17. J. Dauparas, *et al.*, Atomic context-conditioned protein sequence design using

LigandMPNN. *Nat. Methods* **22**, 717–723 (2025).

18. R. Obexer, *et al.*, Emergence of a catalytic tetrad during evolution of a highly active artificial aldolase. *Nat. Chem.* **9**, 50–56 (2017).

19. R. Obexer, *et al.*, Active site plasticity of a computationally designed retro-aldolase enzyme. *ChemCatChem* **6**, 1043–1050 (2014).

20. Y. Kipnis, *et al.*, Design and optimization of enzymatic activity in a de novo β-barrel scaffold. *Protein Sci.* **31**, e4405 (2022).

21. S. Bjelic, *et al.*, Exploration of alternate catalytic mechanisms and optimization strategies for retroaldolase design. *J. Mol. Biol.* **426**, 256–271 (2014).
